# Presumed cancer‐associated retinopathy (CAR) mimicking Sudden Acquired Retinal Degeneration Syndrome (SARDS) in canines

**DOI:** 10.1111/vop.12853

**Published:** 2020-12-27

**Authors:** Sinisa D. Grozdanic, Tatjana Lazic, Helga Kecova, Kabhilan Mohan, Grazyna Adamus, Markus H. Kuehn

**Affiliations:** ^1^ Department of Veterinary Clinical Sciences College of Veterinary Medicine Iowa State University Ames IA USA; ^2^ Animal Eye Consultants of Iowa Hiawatha IA USA; ^3^ TL VetPath International Consultants Hiawatha IA USA; ^4^ Department of Ophthalmology Oregon Health & Science University Portland OR USA; ^5^ Department of Ophthalmology and Visual Sciences Roy J. and Lucille A. Carver College of Medicine University of Iowa Iowa City IA USA; ^6^ Center for the Prevention and Treatment of Visual Loss Iowa City VA Health Care System Iowa City IA USA

**Keywords:** cancer, immunity, microarray, retina, retinopathy, SARDS

## Abstract

**Objective:**

To describe functional and structural features of presumed cancer‐associated retinopathy (CAR) mimicking sudden acquired retinal degeneration syndrome (SARDS) in dogs and describe treatment outcomes.

**Animals:**

Subjects were 17 dogs from 8 eight US states and Canada diagnosed with SARDS or immune‐mediated retinitis (IMR) by 12 ophthalmologists. Nine eyes from seven deceased patients were used for microarray (MA), histology, or immunohistochemical (IHC) analysis.

**Procedures:**

Dogs underwent complete ophthalmic examination, including retinal photography, optical coherence tomography (OCT), chromatic pupil light reflex testing (cPLR), and electroretinography (ERG), in addition to complete systemic examination. Histology, microarray, and IHC analysis were performed in CAR retinas to evaluate histological and molecular changes in retinal tissue.

**Results:**

None of the patients evaluated satisfied previously established criteria for diagnosis of SARDS (flat ERG+ no red – good blue PLR), and all were diagnosed with IMR. All patients were diagnosed with a cancer: meningioma (24%), sarcoma (18%), pituitary tumor (12%), and squamous cell carcinoma (12%), other (34%). Median survival time was 6 months from diagnosis (range 1‐36 months). Most frequent systemic abnormalities were as follows: proteinuria (78%); elevated liver enzymes (47%); and metabolic changes (PU/PD, polyphagia – 24%). Immunosuppressive therapy resulted in the reversal of blindness in 44% of treated patients, with 61% of all treated patients recovering and/or maintaining vision. Median time for preservation of vision was 5 months (range 1‐35 months).

**Conclusions:**

Observed changes are highly suggestive of immune‐mediated damage in IMR‐CAR eyes. A relatively high percentage of patients with CAR responded positively to immunosuppressive therapy.

## 1 INTRODUCTION

Sudden acquired retinal degeneration syndrome (SARDS) is recognized as one of the most frequent irreversible causes of blindness in the canine population. It is characterized by sudden‐onset blindness, completely extinguished retinal electrical responses, and the properties of abnormal chromatic pupil light reflex (cPLR) (no red PLR – good blue PLR).^1^, ^2^, ^3^ It has been theorized that SARDs is an autoimmune disease similar to autoimmune retinopathies in humans (AIR), with a strong retinal autoantibody component.^4^, ^5^, ^6^, ^7^, ^8^

Autoimmune retinopathies (AIR) are rare but devastating autoimmune diseases in humans, characterized by the sudden onset of severe visual deficits (or complete blindness), extinguished retinal electrical activity, relatively normal fundus appearance, and presence of serum retinal autoantibodies.^9^, ^10^, ^11^ AIRs can develop as a form of paraneoplastic syndrome (ie, CAR: cancer‐associated retinopathy; MAR: melanoma‐associated retinopathy), or in the absence of cancer (nonparaneoplastic autoimmune retinopathies: npAIR).^12^, ^13^, ^14^, ^15^ Patients suffering from npAIR can share features of CAR and MAR; however, it is believed that this retinal disease does not develop as a direct result of a cancer.^13^

Autoantibodies (autoAbs) against retinal antigens are considered to be the primary cause of pathology in CAR and npAIR.^15^, ^16^, ^17^, ^18^ Anti‐retinal autoAbs have also been implicated in the pathology of macular degeneration,^17^, ^19^, ^20^, ^21^ retinitis pigmentosa,^20^, ^21^, ^22^ and glaucoma.^23^, ^24^ In these diseases, however, it is not clear whether the antibody production precedes the retinal disease, or the immune reactivity is a consequence of the retinal degenerative process and exposure of degenerating retinal elements to the immune system.^25^ The lack of studies addressing detailed histology and molecular analysis of retinal tissue from CAR human patients further contributes to the relatively poor understanding of mechanisms associated with functional and structural retinal damage.^26^, ^27^, ^28^

While there is an extensive body of literature describing the functional and morphological properties of CAR and MAR in humans,^14^, ^27^, ^29^, ^30^, ^31^ a description of CAR is lacking in the veterinary ophthalmology literature. A single review manuscript describes the features of possible immune‐mediated retinitis (IMR)—CAR in canine patients.^2^ The principal purpose of this study was to describe the clinical, morphological, and molecular properties of presumed CAR in canine patients. Furthermore, due to the extreme similarity between the clinical presentation of SARDS and CAR, the second goal was to describe potential diagnostic parameters that could be used to differentiate between the two conditions. Finally, we evaluate the success of different medical treatment strategies for canine CAR, with a particular focus on a completely novel therapy—the intravitreal application of intravenous immunoglobulin (IVIg).

## 2 MATERIALS AND METHODS

All studies were conducted in accordance with the ARVO Statement for Use of Animals in Ophthalmic and Vision Research. Procedures were approved by the Iowa State University Institutional Committee on Animal Care with protocol numbers: 2‐07‐6307‐K and 5‐07‐6362‐K, while clients pursuing treatment in private hospitals provided a signed consent form for diagnostic and treatment options, including intravitreal IVIg treatment, and possible risks and complications associated with intravitreal injections. A total of 17 canine clinical patients were evaluated for SARDS in the period between September 2006 and April 2017 (Tables 1, 2 and 3). Patients were from eight US states and Canada and were diagnosed with SARDS or immune‐mediated retinitis (IMR) by eight different ophthalmologists. Sixteen patients were diagnosed with a malignant tumor prior to or at the time of development of visual problems, while 1 was diagnosed with malignant tumor 24 months after initial development of blindness.

**Table 1:** Breed, age, and sex distribution of CAR patients with geographic location and clinical findings at the initial examination

	Breed	State	Sex	Age (y)	Duration of vision loss (d)	ERG	cPLR	Fundus changes (indirect ophthalmoscopy)
1	Golden Retriever (OCT, MA‐OD and OS, IHC, H, sAb, vAb)	IA	SF	9	30 (gradual progressive)	Primary ophthalmologist: normal (a‐wave 83 µV, b‐wave 144 µV, flicker not performed) Secondary ophthalmologist: severe reduction of scotopic and flicker responses (a‐wave 41 µV, b‐wave 55 µV, flicker 14 µV, Figure 9A)	NR‐GB	Initially WNL, later pONH, VA
2	Chow Chow (MA‐OD and OS, IHC, H, sAb)	IA	CM	8	Night vision loss for 3 y	Flat	NR‐DB (10‐8 mm)	HR‐PV; HR‐F; PEL, PRE
3	Border Collie (OCT, H)	CA	CM	5	Depth perception issues since age of 2, decreased vision for 2 mo	Primary ophthalmologist 20 µV scotopic b‐wave amplitudes Secondary ophthalmologist normal amplitudes OU (a‐wave 43 µV, b‐wave 130 µV, Figure 9C)	NR‐DB (10‐6)	pONH, VA, HPS, HR‐PV
4	Boxer (H)	IA	SF	7	Night vision loss, depth perception issues for 1 mo	Normal full‐field, decreased flicker (flicker b‐wave 44 µV)	NR‐GB OD; NR‐DB OS	WNL
5	Eskimo (OCT, H)	CA	CM	4.5	1 mo sudden onset of blindness	Flat	NR‐NB	PE OU
6	Miniature Pinscher (OCT, sAb, H, IHC)	TN	CM	7	7 d	Absent rod b‐wave, decreased scotopic (rod‐cone) b‐wave, decreased in cone and flicker, absent pattern ERG, decreased multifocal ERG (for values see Figure 1C)	DR OU (10‐7 OD, 10‐9 OS), GB OD (10‐3), DB OS (10‐7)	VA
7	Brittany Spaniel (sAb, H)	WA	F	9	Depth perception issues and intermittent night vision problems for 6 mo, sudden onset of complete blindness for 2 wk (7‐10 d after vaccination)	Flat (primary ophthalmologist); Secondary ophthalmologist: Scotopic b‐wave 20 µV, bizarre cone response OS – normal cone amplitude b‐wave 36µ V with 30 ms delayed implicit times, scotopic b‐wave 40 µV OD (Figure 1D)	GR‐GB OD, DR‐GB OS (10‐8)	HPS OU
8	Labrador Retriever	IA	M	9	Sudden onset of blindness 4 d prior, normal vision on ophthoexamination	Flat	NR‐DB (10‐6)	pONH, VA, HR‐P, HR‐D, HPS
9	Shih Tzu	MN	CM	11	10 d (blindness after dental cleaning)	Flat	NR‐DB (10‐7)	pONH, VA
10	Boxer	ND	SF	7	14 d sudden onset of blindness	Decreased a and b‐wave (scotopic – 65 µV) OS, normal OD (84 µV), absent flicker OU	NR‐GB	WNL
11	Italian Greyhound	IA	SF	10	14 d, blind OD, night blind OS	Normal scotopic, decreased flicker (flicker b‐wave 47 µV)	NR‐GB	WNL OS, OD cannot be observed
12	Vizsla	IL	M	7	Intermittent vision problems for 90 d, responsive to steroids and cyclosporine	Flat (primary ophthalmologist); 40 µV b‐wave amplitudes OU	NR‐DB (10‐8 OD, 10‐6 OS)	WNL ‐ OD ATR ‐ OS
13	Mixed	MO	CM	9	Decreased vision for 3 mo, primarily dim light; on ophthalmic examination normal	Flat	DR (10‐7)‐ GB	pONH, VA
14	Toy Manchester Terrier	IA	SF	8	Intermittent night vision problems Hx; no vision problems on ophthalmology examination	Normal full‐field, decreased flicker (32 µV)	NR‐DB (10‐5)	WNL
15	Mixed	IA	SF	9	Intermittent night vision problems	N/A	NR‐GB	HR‐D; HR‐PV; HR‐F
16	Dachshund (OCT, sAb)	IA	CM	10	21 d sudden onset of blindness	Flat	GR‐GB	pONH, VA
17	Mixed	IL	CM	14	7 d sudden onset of blindness	Flat	GR‐GB	VA, PRE

Note

Sex: F, female; M, female; SF, spayed female; CM, castrated male; OD, right eye; OS, left eye; OU, both eyes; OCT, optical coherence tomography performed; MA, microarray analysis on retinal tissue performed; sAB, Western blot testing on serum performed; vAB, Western blot testing on vitreal sample performed; IHC, immunohistochemistry on ocular sections performed; H, ocular histology performed.

Legend for chromatic pupil light reflex (cPLR): NR, no red; GR, good red, DR, decreased red; NB, no blue; GB, good blue; DB, decreased blue; 10 (starting pupil diameter in mm before illumination) – 8 (end pupil diameter in mm after illumination).

Legend for fundus changes: pONH, pale optic nerve head; VA, vascular attenuation; HPS, hyperpigmented spot; ATR, altered tapetal reflectivity (“tapetal ridging”); HR, hyper‐reflectivity (HR‐F, focal; HR‐PV, perivascular; HR‐D, diffuse); FH, focal hemorrhage; PE, optic nerve head papilledema; PEL, perivascular exudative lesion; PRE, perivascular retinal edema; WNL, within normal limits; the first column has dog numbers which are identical across Tables 1, 2, and 3. Reference ERG values for our laboratory are >75 µV for full‐field combined rod‐cone response b‐wave and >60 µV for flicker b‐wave response (retinographics unit).

**Table 2:** Laboratory and systemic abnormalities in CAR patients

	PU/PD‐PP‐WG	Serum	Urine	CBC	BP	Allergies/Autoimmune diseases	Other systemic changes
1*	None	↑cholesterol and lipids, ↑ALT	N/A	WNL	N/A	None	Membranous glomerulonephritis (necropsy); liver vacuolar degeneration (glycogen type)
2	None	↑SAP	N/A	WNL	WNL	Atopy	None
3*	None	WNL	WNL	WNL	WNL	Atopy	Pancreatitis 11 mo after initial diagnosis
4	None	WNL	WNL	WNL	WNL	Atopy	None
5	PU/PD	↑cholesterol	Microalbuminuria, proteinuria	WNL	WNL	Atopy	Ataxia, proprioceptive deficits, nystagmus
6	PP, WG for 6 mo	↑ALT, ↑SAP*, ↑Ca^++^ ↑total protein,	Proteinuria, trace of blood	Leukopenia	N/A	Atopy	Membranous glomerulonephritis (necropsy); liver vacuolar degeneration (glycogen type)
7	None	WNL	N/A	WNL	WNL	Atopy	MRI‐mild hydrocephalus, wider and deeper sulci—possible cerebral cortical atrophy, heavy sedated behavior
8	None	WNL	Proteinuria, microalbuminuria	WNL	180‐200	None	None
9	PU/PD, PP 2 mo	↑cholesterol, ↑SAP*	N/A	WNL	N/A	Atopy/food allergies; KCS	None
10	None	↑lipase	N/A	WNL	WNL	None	Pancreatitis
11	None	N/A	N/A	N/A	N/A	Atopy	None
12*	None	↑SAP, ↑cholesterol	Microalbuminuria	WNL	WNL	Atopy	None
13	None	WNL	Microalbuminuria, proteinuria	WNL	185 mm Hg	None	None
14	None	↑SAP, ↑ALT,	N/A	neutrophilia	N/A	Atopy, bacterial skin infections	Small liver
15	None	↑BUN	N/A	WNL	N/A	Atopy	None
16	None	↑SAP	Proteinuria, microalbuminuria	WNL	N/A	Food allergies; allergic bronchitis, KCS	None
17	PU/PD for 1 y	↑SAP, ↑cholesterol, cPLI, ↓T4	Proteinuria	WNL	WNL	None	None

Legend: PU/PD, polydipsia/polyuria; PP, polyphagia; weight gain, WG; SAP, serum alkaline phosphatase; ALT, alanine aminotransferase; BUN, blood urea nitrogen; cPLI, canine pancreatic lipase; Ca^++^, serum calcium; KCS, keratoconjunctivitis sicca (dry eye), N/A, data not available; WNL, within normal limits.

* Treated with systemic steroids prior to laboratory analysis.

**Table 3:** Summary of medical treatment and response to therapy in CAR patients

	Breed	Sex	Age (y)	S (m)	V (m)	Tumor	Medications	Response to therapy
1	Golden Retriever	SF	9 y	6	6	Brain meningioma (pituitary region)*	Initial response to prednisone 1 mg/kg BID PO then worsening, no response to pred + chlorambucil PO; Response to pred and intravitreal IVIg	Visual until death—pulmonary thromboembolism, necropsy
2	Chow Chow	CM	8	36	35	Oral mucosal melanoma 3 y prior, cardiac hemangioma with isolated brain metastasis at time of death*	None	None—euthanized due to neurological symptoms, necropsy
3	Border Collie	F	5	36	30	Brain Meningioma (pituitary region)*	Prednisone + doxycycline PO BID for 3 mo—improved vision and ERG, then decline, Intravitreal IVIg + triamcinolone +systemic prednisone PO—visual for 1 y, then vision decline, then intravitreal steroid (every 6‐8 mo) + low‐dose systemic prednisone PO	Visual in variable degrees for 2.5 y. Last 6 mo blind, on radiation therapy for possible pituitary tumor, necropsy—meningioma
4	Boxer	SF	7	11	0	Brain meningioma (pituitary, chiasmal)*	Intravitreal IVIg + triamcinolone, prednisone 0.5‐1 mg/kg BW PO BID, doxycycline PO BID	Complete vision loss after brief anesthesia, crude visual navigation skills recovered 2 wk after injection, neurological symptoms and euthanasia 11 mo after treatment, necropsy
5	Eskimo	CM	10	1	0	Pituitary carcinoma*	Prednisone 1 mg/kg BW PO BID for 3 wk by primary ophthalmologist—no response	Pituitary carcinoma—necropsy
6	Miniature Pincher	SF	9	3	0	SCC oral (tonsillar), orbital OS, frontal bones*	Prednisone 0.5‐1 mg/kg BW PO BID—3 mo	No response—euthanasia after 3 mo
7	Brittany Spaniel	F	9	12	0	Mammary adenocarcinoma (3 y prior), multiple mammary nodules on ultrasound (FNA inconclusive) at the time of blindness*	Prednisone 0.5‐1 mg/kg BW QD‐BID PO + doxycycline BID PO	No response—euthanasia after 12 mo due to worsening of neurological status
8	Labrador Retriever	M	9	6 LF	6	Testicular carcinoma*	Cyclosporine q24h PO, benazepril, aspirin, amlodipine PO	Normal vision, no episodes of vision loss, lost to follow‐up after 6 mo
9	Shih Tzu	CM	11	3 LF	0	Brain meningioma or glioma	Prednisone 0.5‐1 mg/kg BW	No response—lost to follow‐up after 3 mo
10	Boxer	SF	7	2	2	Pituitary mass	Leflunomide QD, mycophenolate BID, maropitant citrate q48h PO	Vision recovery for 2 mo, then euthanasia due to seizures and worsening of neurological status
11	Italian Greyhound	SF	10	3 LF	3	Ciliary body/choroidal mass OD	None	Lost to follow‐up, phone interview—blind 3 mo post‐diagnosis
12	Vizsla	M	7	6 LF	3	Mast cell tumor 3 y before*	Prednisone 0.5‐1 mg kg BW PO BID	Initial response to therapy for 3 mo, blind after 6 mo
13	Mixed	CM	10	4 LF	4	Shoulder sarcoma or hemangiopericytoma*	Enalapril, aspirin PO	Lost to follow‐up after 4 mo
14	Toy Manchester Terrier	SF	8	1	1	Diffuse SCC*	Systemic antibiotics for bacterial skin infection PO	Euthanasia in 1 mo due to worsening of the systemic status
15	Mixed	SF	9	4	4	Diffuse mast cell tumor with bone marrow involvement*	Prednisone 0.5‐1 mg/kg BW PO	Visual until euthanasia 4 mo after initial diagnosis
16	Dachshund	SF	10	24	16	Muscle sarcoma (24 mo after initial diagnosis of blindness). On initial diagnosis CT, thoracic and abdominal radiographs clean*	Prednisone (1.5 mg/kg BID PO) + doxycycline initially for 1 mo – no response, intravitreal IVIg + systemic prednisone 1 mg/kg QD PO for 10 d, then q48h response, cyclosporine QD PO later (when blind 2nd time—no response)	Visual for 16 mo after treatment, then complete blindness (prednisone discontinued)
17	Mixed	SF	14	6	2	Spindle cell sarcoma removed 2 y prior to blindness*	Prednisone 1mg/kg BW BID PO + doxycycline recovery of vision in dim and bright light—2 mo later bilateral retinal detachments with subretinal macrophage population (subretinal aspirate)	Blindness 2 mo after initiating treatment

Abbreviations: SF, spayed female, CM, castrated male, LF, lost to follow‐up, OS, left eye, SCC, squamous cell carcinoma, BID, drug application 2 times per day, QD, drug application once daily. Intravitreal IVIg (0.2ml, Gammaguard 10%, Baxter International INC, Deerfield, IL) was used for treatment of clinical patients.

* Diagnosis confirmed by histopathology.

Nine eyes were collected for histology, microarray (MA), or immunohistochemistry (IHC) analysis from seven IMR‐CAR patients (Table 1, patient numbers 1, 2, 3, 4, 5, 6, and 7) euthanized within 2 months to 5 years of IMR‐CAR diagnosis. One half of the right and one half of the left eye were collected from 2 IMR‐CAR patients for microarray analysis (patients number 1 and 2), while remaining halves from these patients were used for histology or IHC analysis. An additional 10 control eyes from healthy control dogs (Beagle, SF, 6 y old) without evidence of ocular abnormalities were used for microarray and immunohistochemistry analysis. Serum samples from 10 glaucomatous Basset Hounds from the Iowa State University colony and 7 healthy Beagles were used for the serum retinal autoantibody detection.

### 2.1 Diagnosis of the presumed CAR phenotype

Previously published criteria for diagnosis of SARDS were used to characterize all patients: completely extinguished electroretinography (ERG) response (flat ERG), in combination with no pupil light reflex after red light illumination (NR) and good pupil light reflex after blue light illumination (GB).^2^ All CAR patients and healthy control dogs received a complete ophthalmic examination: slit‐lamp biomicroscopy, indirect ophthalmoscopy, tear production and intraocular pressure evaluation, and basic neuro‐ophthalmology evaluation (palpebral and corneal reflex, ocular motility evaluation). Menace, dazzle, PLRs, and visual maze testing was performed to evaluate the status of the visual system.

### 2.2 Systemic organ function evaluation

All patients also underwent complete cell blood count and serum chemistry, urine analysis (10 of 17, all samples collected via cystocentesis), systolic blood pressure evaluation (10 of 17, systolic blood pressure was evaluated using an ultrasonic Doppler flow detector; Model 811‐L, Parks Medical Electronics Inc, Las Vegas, NV, USA), and thoracic and abdominal radiographs (17 of 17). In 7 of 17 patients, brain computerized tomography (CT) or magnetic resonance imaging (MRI) was performed. Abdominal and thoracic radiographs, and brain CT images were reviewed by board‐certified radiologists and residents at the institutions where imaging was performed. Only head CT/MRI imaging was pursued (no thorax or abdomen CT imaging was conducted).

### 2.3 Functional and structural retinal evaluation in vivo

The vision was evaluated by observing following parameters in dim and bright light conditions: menace, cotton ball tracking, and visual navigation in the maze test. The presence of one or multiple positive visual responses was marked as presence of the vision.

Pupil light reflex (PLR), chromatic pupil light reflex (cPLR), fundus photography, and electroretinography (ERG) were performed in clinical patients and experimental dogs as described in previous studies.^2^, ^32^, ^33^, ^34^ Optical coherence tomography (OCT) analysis in canine patients was performed as previously described.^32^

### 2.4 General anesthesia protocol for ERG and OCT testing

Dogs were anesthetized for ERG and OCT testing as previously reported.^5^, ^32^, ^33^

### 2.5 Electroretinography (ERG)

Electroretinography (full‐field and pattern routines) was used to evaluate retinal function in IMR‐CAR dogs (n = 16). A Roland Consult ERG system (Brandenburg, Germany) was used for pattern ERG routines and the International Society for Clinical Electrophysiology of Vision (ISCEV) ERG routines under general anesthesia, while retinographics ERG system (Norwalk, CT) was used for ERG testing in nonanesthetized and nonsedated patients. Both ERG systems were used to deliver light stimuli and collect signals from the lens electrode for full‐field ERG recording routines, as previously reported.^2^, ^5^, ^32^

### 2.6 Multifocal ERG recording protocol

The mfERG routine was recorded after a 15‐minute period of dark adaptation using the VERIS Science system (Edi Inc, Redwood City, CA, USA) with fundus camera for projection of the stimulus pattern in one clinical patient. A pediatric bipolar Burian‐Allen electrode (Hansen Ophthalmic Development Laboratory, Iowa City, IA) was used to record the mfERG routine. Since a fundus camera with projecting stimuli was used for mfERG recordings, the electrical activity was recorded from only one eye at a time. The reference electrode was positioned in the forehead region between the two eyes, while the ground electrode was placed on the back of the head (occipital region). Both electrodes were inserted subcutaneously.

The first‐order response of mfERG was analyzed in CAR patients and control dogs. The stimulus routine consisted of 37 black and white hexagons with temporal settings: frequency = 75 Hz, M sequence = 15, Frames = 1, number of segments = 16, pre‐exposure time = 1 s, samples per frame = 16. Low cut amplifier frequency was set at 3 Hz, while high cut amplifier frequency was set at 300 Hz.

### 2.7 Fundus photography

Fundus photography was performed using the RetCam Fundus Camera system (Massie Research Laboratories, Pleasanton, CA) as previously reported.^5^

### 2.8 Optical coherence tomography (OCT)

Optical coherence tomography and analysis of different retinal layer thickness were performed using Heidelberg Spectralis SD‐OCT unit as previously reported.^5^, ^33^ The following scans were obtained: horizontal volume scan through *area centralis* (within the visual streak; located dorsal‐temporally from the optic nerve head) in the superior‐temporal (tapetal) retina, with a corresponding volume scan in the ventrotemporal (nontapetal) retina. Additional horizontal volume scans were performed based on the funduscopic evidence of possible retinal lesions with a focus on hyperpigmented, hyper‐reflective, and potentially exudative lesions. The goal of scans was to detect possible retinal lesions that were not evident on a regular ophthalmoscopy examination, as previously reported for SARDS dogs.^5^

### 2.9 Histological and immunohistochemical (IHC) analysis

Six eyes removed from four euthanized IMR‐CAR patients, and six additional eyes from healthy beagles with previously performed ophthalmic examination to rule out presence of the ocular disease were fixed and prepared for histology analysis as previously described.^5^, ^35^

Histological and immunohistochemical analysis on six IMR‐CAR eyes from three patients (collected 1‐35 months after IMR‐CAR diagnosis) and six untreated healthy beagle eyes were performed as previously reported using anti‐CD3 (T‐lymphocyte marker); anti‐CD79 (B‐lymphocyte marker); anti‐CD11b (microglia marker); and a cocktail of IgG, IgM, and IgA for detection of immunoglobulin‐producing plasma cells as previously described.^5^, ^35^

### 2.10 Detection of serum and intravitreal retinal autoantibodies—Western blot analysis

Initial screening of dog IMR‐CAR sera (n = 5), IMR‐CAR vitreal sample (n = 1), control healthy dog canine sera (n = 7), and banked frozen primary glaucoma dog sera (n = 10) was performed using dog retinal proteins that were extracted from retinas as previously described.^36^

### 2.11 Microarray (MA) analysis

Microarray analysis on canine retinal tissue was performed as previously reported.^5^ Briefly, immediately after enucleation eyes were preserved in RNAlater (Thermo Fisher). Retinas were then dissected, and RNA was extracted and hybridized to Canine Genome 2.0 arrays (Thermo Fisher) following the manufacturer's instructions. Data were obtained from two eyes of an untreated IMR‐CAR patient, two of a IMR‐CAR patient treated with intraocular IVIG who responded to therapy, three from three patients with SARDS (previously published SARDS microarray dataset^5^ was used for comparison with IMR‐CAR microarray data), and two from two healthy control dogs without evidence of ocular abnormalities.

### 2.12 Statistical analysis

Obtained microarray data were normalized using the robust multi‐array average (RMA) module in R (version 3.6).^37^ Probesets that yielded low expression values in all samples were removed from the dataset. Hierarchical clustering and PCA (principal component analysis) were carried out using ClustVis software (version 2.0).^38^ Functional assignments were generated using DAVID (database for annotation, visualization, and integrated discovery, v6.8)^39^ and STRING.^40^

Calculation of mean, standard deviation, and medial value and paired t test was performed with commercial software (Prism, version 5.0; GraphPad, San Diego, CA). Value of *P* < .05 was considered statistically significant.

## 3 RESULTS

### 3.1 Patient population

Subjects were 17 dogs from eight US states and Canada diagnosed with SARDS or IMR based on history and results of ophthalmology and general examination by eight ophthalmologists (Tables 1 and 2).

Male patients were more prevalent (10 of 17 patients, 59%) than females (41%, Table 1). The mean age for the population was 8.4 ± 2.2 years (mean ± SD, median value = 9 y, range 4.5‐14 y). There was no specific breed predisposition (Table 1). Large and small breed dogs were equally represented with 41% large breed dogs, 41% small breed dogs, and 18% mid‐size breed dogs (Table 1). On clinical presentation, 59% of patients were completely blind (10 of 17), 17% were intermittently blind (3 of 17), and 24% (4 of 17) patients were night blind (Table 1).

Sixteen patients were diagnosed with a malignant tumor prior to or at the time of visual problem onset, while one was diagnosed with a malignant tumor 24 months after initial development of blindness (Table 3). The most frequent tumors were as follows: meningioma (n = 4), sarcoma (n = 3), pituitary tumor (n = 2), and squamous cell carcinoma (n = 2). Median survival time was 6 months from diagnosis (range 1‐36 months). All patients were deceased by May 2017 (Table 3).

### 3.2 Functional analysis

**Figure 1:**
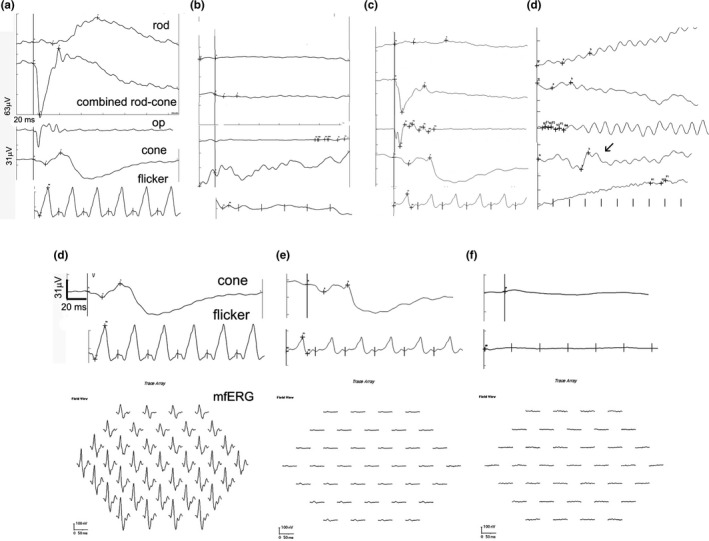
ERG characterization of CAR‐IMR patients: (A) ERG tracings from a control healthy dog for different International Society for Clinical Electrophysiology of Vision (ISCEV) routines; (B) completely extinguished ERG function in all tested routines (mix breed, CM, 9 y old, Table 1, patient no. 13); (C) completely extinguished low light rod response, reduced combined rod‐cone response, with some reduction in cone function (Miniature Pinscher, CM, 7 y old, Table 1, patient no. 6); (D) completely extinguished rod function, with minimal rod‐cone response, and bizarre cone responses (increased implicit time for the full‐field cone response and absent flicker response) in IMR‐CAR canine patient with mammary adenocarcinoma (Springer Spaniel OS, F, 9 y old, patient, Table 1, no. 7). (E) The International Society for Clinical Electrophysiology of Vision (ISCEV) ERG cone routines and mfERG routine tracings from a control healthy dog; (F) In this patient, a decrease in the ISCEV full‐field cone response and flicker cone responses was observed; however, disproportionately severe suppression of the mfERG responses was observed most likely indicative of the primary central retina (area centralis) cone function suppression considering that the mfERG primarily evaluates central retina cone function (Miniature Pinscher, CM, 7 y old, Table 1, patient no. 6); (G) completely extinguished full‐field ERG function in SARDS canine patient (not part of this study) shown as an example for comparison

**Figure 2:**
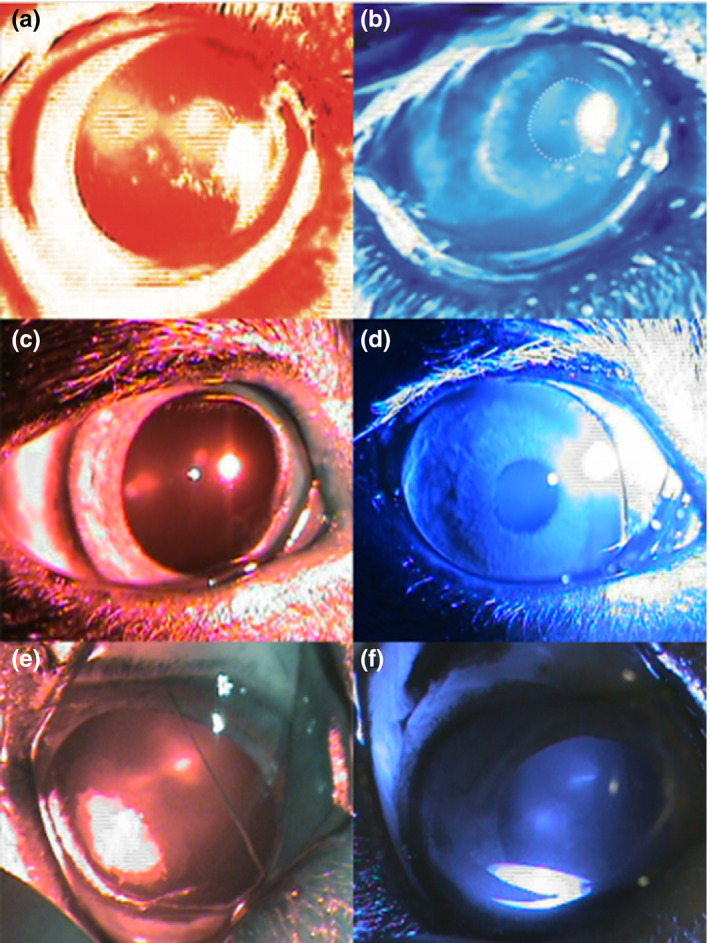
Chromatic pupil light reflex (PLR) testing in IMR‐CAR patients: (A) and (B) Lack of PLR after red light illumination and good PLR after blue light illumination (Golden Retriever, spayed female ‐ SF, 9 y old, Table 1, patient no. 1); (C) and (D) poor but present PLR after red light illumination and good PLR after blue light illumination (Miniature Pinscher, CM, 7 y old, Table 1, patient no. 6); (E) and (F) lack of PLR after red light illumination and poor, but present PLR after blue light illumination (Chow Chow, castrated male—CM, 8 y old, Table 1, patient no. 2)

A total of 56% (9 of 16) of patients had completely extinguished retinal electrical responses (flat ERG), 18.7% (3 of 16) had normal full‐field responses but decreased or absent flicker ERG responses, 18.7% (3 of 16) had decreased full‐field responses and flicker ERG responses, while one had near‐normal full‐field scotopic and photopic responses, but absent rod and pattern ERG responses, and suppressed mfERG responses (Table 1, Figure 1). Chromatic pupil light reflex testing revealed completely absent cPLR after red light illumination and normal cPLR after blue light illumination in 23.5% of patients (4 of 17; Table 1, Figure 2). Two patients had completely normal cPLR responses (both had completely extinguished ERG responses), one had completely absent cPLR response after red and blue light illumination, while the rest had different combination of cPLR deficits in one or both eyes, with no PLR after red light illumination and decreased PLR response after blue light illumination being the most common type of deficit (total of seven eyes). None of these patients had the classic combination of SARDS responses: flat ERG combined with no red‐good blue cPLR (Table 1).

### 3.3 Fundoscopic findings

**Figure 3:**
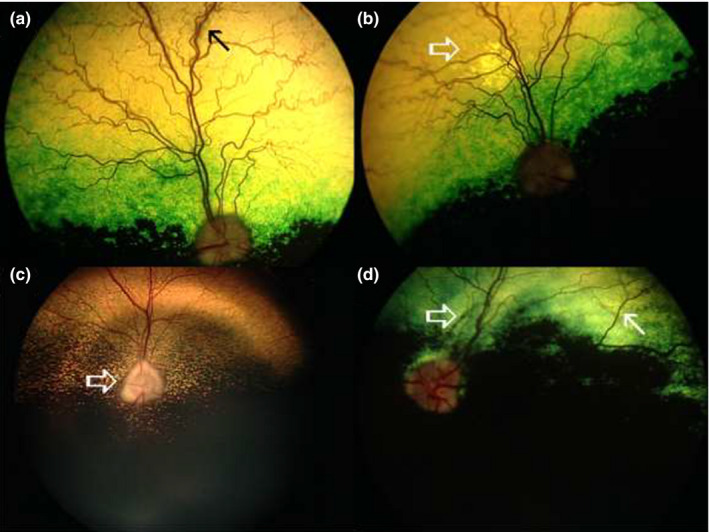
Fundus appearance in CAR‐IMR patients: (A) Perivascular retinal edema and exudative lesion, indicated by arrow (Chow Chow, castrated male—CM, 8 y old, Table 1, patient no. 2, OS); (B) perivascular retinal degenerative changes presenting as perivascular zones of hyper‐reflectivity (arrow) in Chow Chow (CM, 8 y old, Table 1, patient no. 2, OD); (C) pONH appearance (arrow) (Golden Retriever, SF, 9 y old, Table 1, patient no. 1); (C) hyperpigmented peripapillary linear lesion (open arrow) and perivascular hyper‐reflectivity in Miniature Pinscher (CM, 7 y old, Table 1, patient no. 6)

Fundoscopic evaluation revealed presence of various changes. The most frequently observed fundus change was vascular attenuation (VA) resulting in decreased diameter of blood vessels, and sporadic loss of tertiary retinal vein branches, which was observed in 41% of patients (7 of 17; Table 1, Figure 3). In addition, pale optic nerve head appearance (pONH) was observed in 23.5% of patients (4 of 17; Table 1, Figure 3). Normal fundus was observed in 23.5% of patients (4 of 17), while an additional two had one eye with normal fundus appearance. Altered tapetal reflectivity, perivascular hyper‐reflective lesions, chorioretinal scars, hyperpigmented spots, and retinal edema could be observed in 23.5% of patients (4 of 17). One patient had bilateral optic nerve head papilledema.

### 3.4 Optical coherence tomography (OCT) and retinal histology analysis

**Figure 4:**
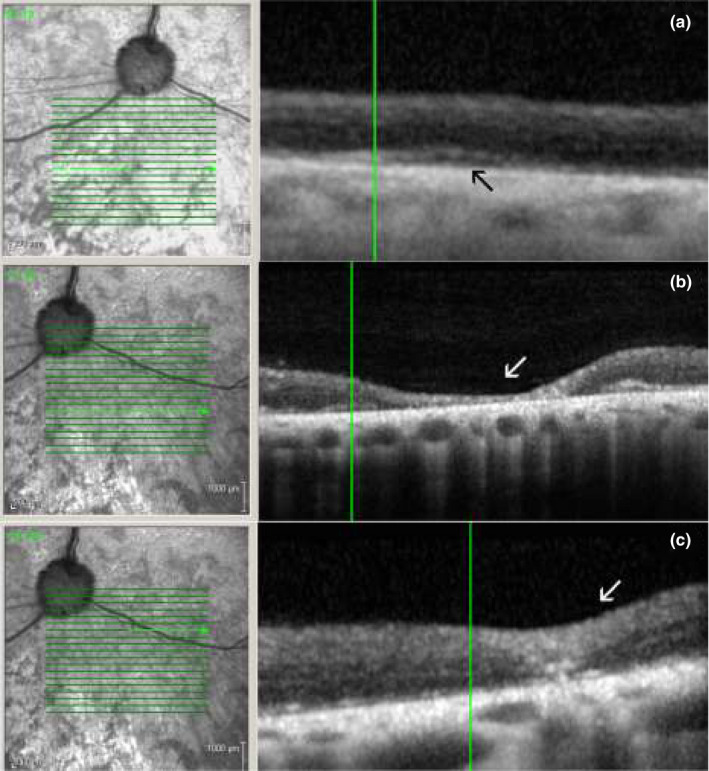
OCT characterization of CAR‐IMR eyes. A, Focal microretinal detachments (RD) were not detectable during fundus evaluation or in fundus photography; however, the RDs could be observed using OCT imaging in CAR‐IMR eyes. Infrared fundus image of the retinal region evaluated by OCT revealed some vascular attenuation but no RD. Arrow points to the region where focal RD was detected using OCT (Border Collie, CM, 5 y, Table 1, patient no. 3). B, OCT of CAR‐IMR retina shows focal zone with the complete loss of retinal structure (arrow) in the same patient (Border Collie, CM, 5 y, Table 1, patient no. 3). C, OCT of CAR‐IMR retina shows focal perivascular zone with the chorioretinal scar in the same patient (Border Collie, CM, 5 y, Table 1, patient no. 3). In all images, there is evident loss of the photoreceptor inner segment and outer segment stratification

**Figure 5:**
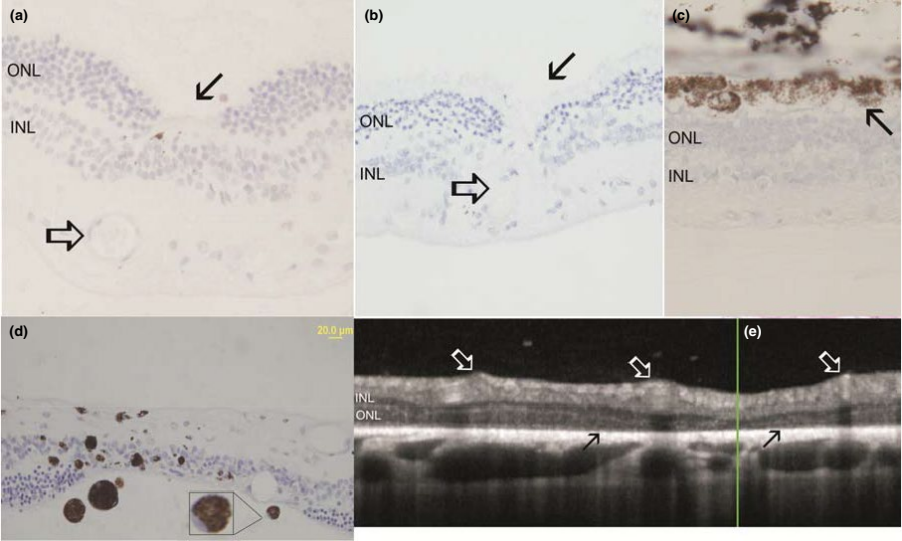
Focal outer nuclear layer loss and diffuse loss of the outer nuclear layer (ONL) are frequently observed in CAR‐IMR patients. A and B, Retinal histology micrograph shows focal photoreceptor loss (arrow) in the close proximity to blood vessels (open arrow). Image A is from Miniature Pinscher (CM, 7 y old, Table 1, patient no. 6). C, Retinal histology micrograph shows outer nuclear layer (ONL) and outer segment loss, with reactive retinal pigment epithelium changes (arrow). Image C is from Golden Retriever (SF, 9 y old, Table 1, patient no. 1). D, Retinal histology micrograph shows diffuse retinal architecture loss, with infiltration of pigmented macrophages (outlined image shows zoomed appearance of one pigmented macrophage with typical peripheral nucleus position). Images D and E are from Golden Retriever (SF, 9 y old, Table 1, patient no. 1). E, OCT of the retina shows focal photoreceptor loss and diffuse loss of the IS‐OS organization (dark arrows) in perivascular spaces (white arrows point to blood vessels). The loss of the photoreceptor inner segment and outer segment stratification is evident (ONL, outer nuclear layer; INL, inner nuclear layer)

Optical coherence tomography analysis was performed in five patients and comparison to fundus images revealed that zones of hyper‐reflective appearance were associated with focal retinal structural photoreceptor loss (Figures 4 and 5). Diffuse loss of the inner segment‐outer segment (IS‐OS) photoreceptor organization was evident (Figures 4 and 5). Zones of perivascular exudative lesions (PEL) were associated with exudative retinal detachments, retinal edematous, and cystic changes or retinal perivascular thinning (Figures 4 and 5). Histology analysis revealed the presence of photoreceptor loss, which had a focal perivascular nature in some cases, activation of retinal pigment epithelium, and active macrophage appearance (Figure 5).

### 3.5 Systemic organ changes

Analysis of systemic changes (increased appetite/polyphagia, weight gain, polydipsia/polyuria) was performed based on historical information obtained from owners, referring veterinarians, and a review of patient medical records. Clinical signs of poldypsia/polyuria (PU/PD), polyphagia (PP), and weight gain (WG) were observed in 23.5% of patients (4 of 17; Table 2). Evaluation of serum chemistry revealed elevation of serum alkaline phosphatase (SAP) and/or alanine aminotransferase (ALT) in 47% of patients (8 of 17).

Serum analysis of IMR‐CAR patients revealed elevated cholesterol levels in 23.5% of patients (4 of 17). Urine analysis revealed proteinuria/microalbuminuria in 77.7% (7 of 9; urine analysis data were missing for eight patients; Table 2). History of pancreatitis with abnormal cPL or elevated sPL, or elevated serum lipase was present in 30% (3 of 10; this was not evaluated in 7). Serum calcium was elevated in 5% of patients (1 of 17). Blood pressure evaluation revealed systemic hypertension in 20% of patients (2 of 10; this was not evaluated in 7; Table 2). Systemic hypertension was classified as systolic blood pressure equal or higher than 160 mm Hg. Historical presence of allergic and autoimmune diseases was documented for 70.5% of patients (12 of 17, Table 2). Atopy (91.6%, 11 of 12), food allergies (16.6%, 2 of 12), and dry eye disease (16.6%, 2 of 12) were the most frequently observed allergic/autoimmune diseases (Table 2).

### 3.6 IHC characterization of IMR‐CAR retinas

**Figure 6:**
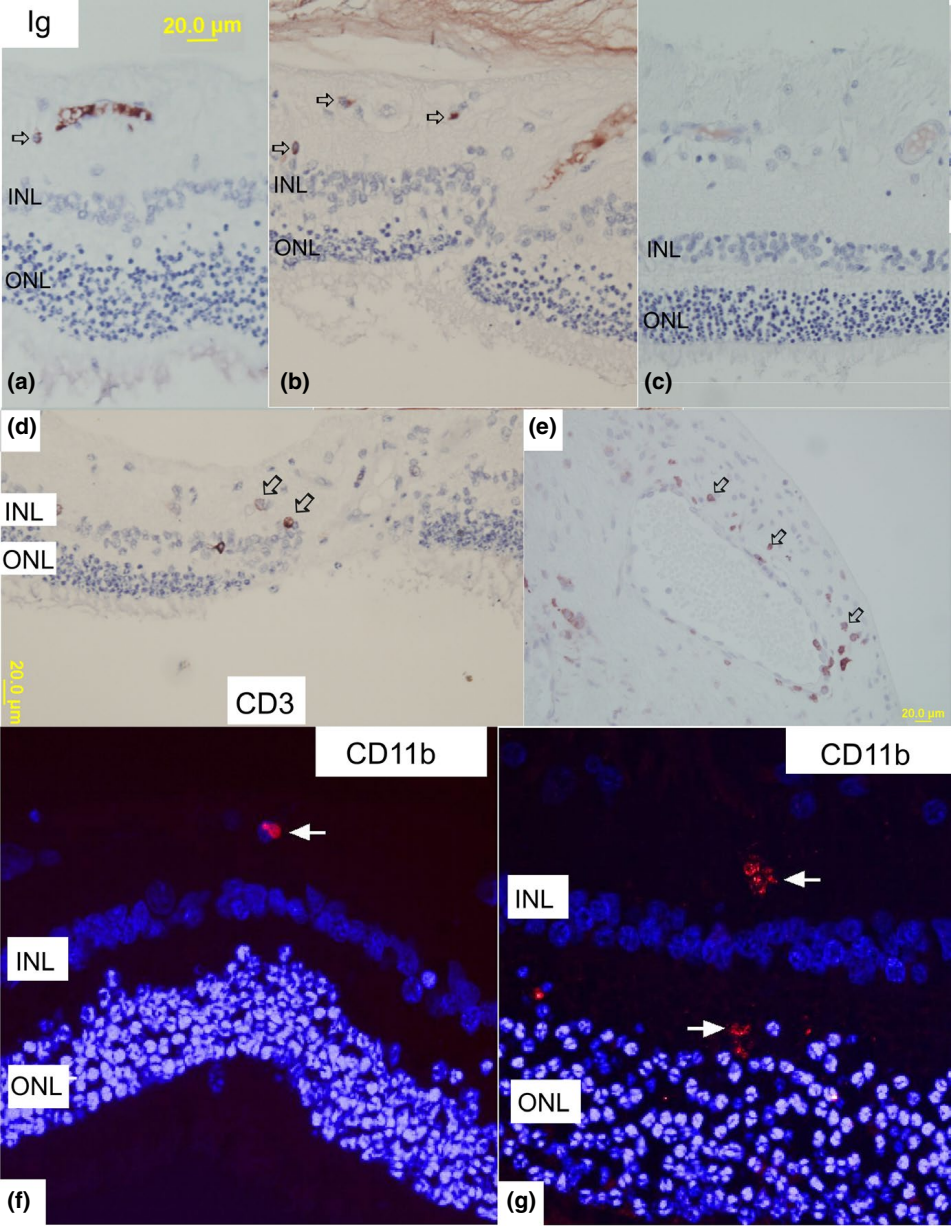
IHC analysis of immune cells in CAR‐IMR retinas collected within 35 and 3 mo after onset of visual deficits. A, IHC staining shows immunoglobulin (Ig) positive cells with typical plasma‐cell morphology (large cells with smaller nucleus and rather extensive cytoplasm staining shows presence of cells in the inner retina (open arrows). Extensive staining is present in the blood vessel lumen (Chow Chow, CM, 8 y old, Table 1, patient no. 2). B, Cells with plasma‐cell morphology (open arrows) are visible primarily in the inner retinal layers in the Miniature Pinscher (CM, 7 y old, Table 1, patient no. 6). C, IHC staining did not show positive stain for cells, just blood vessel lumen in this specimen (Golden Retriever, SF, 9 y old, Table 1, patient no. 1). D, Anti‐CD3 antibody (T‐cell marker) staining shows presence of sporadic T lymphocytes in the focal region of retinal damage (open arrows point to some and not all immunoreactive cells). (Chow Chow, CM, 8 y old, Table 1, patient no. 2). E, Anti‐CD3 antibody (T‐cell marker) staining shows presence of numerous T lymphocytes in the perivascular space of the optic nerve head (open arrows point to intensively labeled cells with typical lymphocyte morphology—smaller cells with large nuclei almost completely filling the cellular space). Image B is from Miniature Pinscher (CM, 7 y old, Table 1, patient no. 6). F, Healthy (control) canine retina shows sporadic presence of the quiescent microglial cell (round cell with regular margins) labeled with anti‐CD11b antibody (white arrow); (G) Canine CAR‐IMR retina shows more prominent microglial cell numbers and phenotype (cells with irregular margins marked by white arrows) in the inner and outer plexiform layers, and outer nuclear layer (Miniature Pinscher (CM, 7 y old, Table 1, patient no. 6)). Legend: ONL, outer nuclear layer; INL, inner nuclear layer

In order to characterize the presence of immune cells in retinal tissue, antibodies against T‐cell marker (anti‐CD3), B‐cell marker (anti‐CD79), microglial marker (CD11b), and plasma‐cell marker (anti‐Ig cocktail) were used. Immunostaining analysis showed focal presence of immune cells (Figure 6), which were usually located in perivascular spaces and regions of focal retinal damage. T cells seemed to be the predominant cell population identified in retinas of deceased patients (Table 4). We did not perform IHC grading of evaluated retinas, since retinal sections frequently had just a few focal regions of cellular infiltrates per evaluated slide.

**Table 4:** Presence of immune cells in canine CAR retinas

	CAR‐IMR	CTRL
T cell (CD3)	100% (3 of 3)	0% (0 of 6)
B cell (CD79)	0% (0 of 3)	0% (0 of 6)
Plasma cell (Ig cocktail)	66.6% (2 of 3)	0% (0 of 6)
Microglia (CD11b)—active	66.6% (2 of 3)	16% (1 of 6)

Note

CD3^+^ T cells were the most frequently observed cell population infiltrating CAR canine retinas (freshly collected ocular tissue was available from dogs 1, 2, and 6—Table 1).

Abbreviations: CAR, cancer‐associated retinopathy; CTRL, healthy control dogs.

### 3.7 Retina serum autoantibodies—Western blot analysis

**Figure 7:**
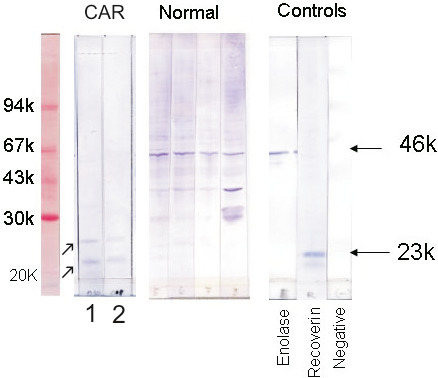
Western blotting analysis of anti‐retinal autoAbs present in sera (gel 1) and vitreous (gel 2) of a CAR‐IMR patient (Patient 1, Table 1, meningioma) before treatment and in control healthy dogs (Normal). Arrows point to bands at 20 and 24 kD molecular weight in the patient sera and vitreal sample (collected prior to intravitreal IVIg injection). Positive control antibodies reacted with enolase and recoverin, and negative control did not contain primary antibodies (just secondary antibodies)

Western blotting analysis revealed the presence of serum anti‐retinal autoAbs in 80% of tested canine IMR‐CAR patients (Table 5, Figure 7). Serum samples of glaucomatous dogs were positive for autoAbs in 100% of cases, while 70% of control healthy dogs were seropositive for anti‐retinal autoAbs. Analysis of the antibody reactivity revealed similarities in retinal antigen recognitions between serum samples of healthy controls, glaucoma dogs, and IMR‐CAR dogs.

**Table 5:** Presence of retinal autoantibodies in the serum of healthy dogs (CTRL), dogs with primary glaucoma, and dogs with CAR

Group	% of dogs with positive WB bands	Band molecular weight (kDa)
CAR	80% (4 of 5)	20, 23, 30, 39, 45, 46, 64, 67, 120
Glaucoma	100% (10 of 10)	30, 35, 36, 38, 40, 44, 46, 50, 60, 64, 67, 70
CTRL	70% (7 of 10)	25, 30, 34, 46, 67

Note

Western blot analysis showed that some autoAbs were shared among all groups (30, 46, 67 kDa). Dogs with glaucoma had the highest variety of retinal autoAbs.

### 3.8 Microarray analysis of canine IMR‐CAR retina

**Figure 8:**
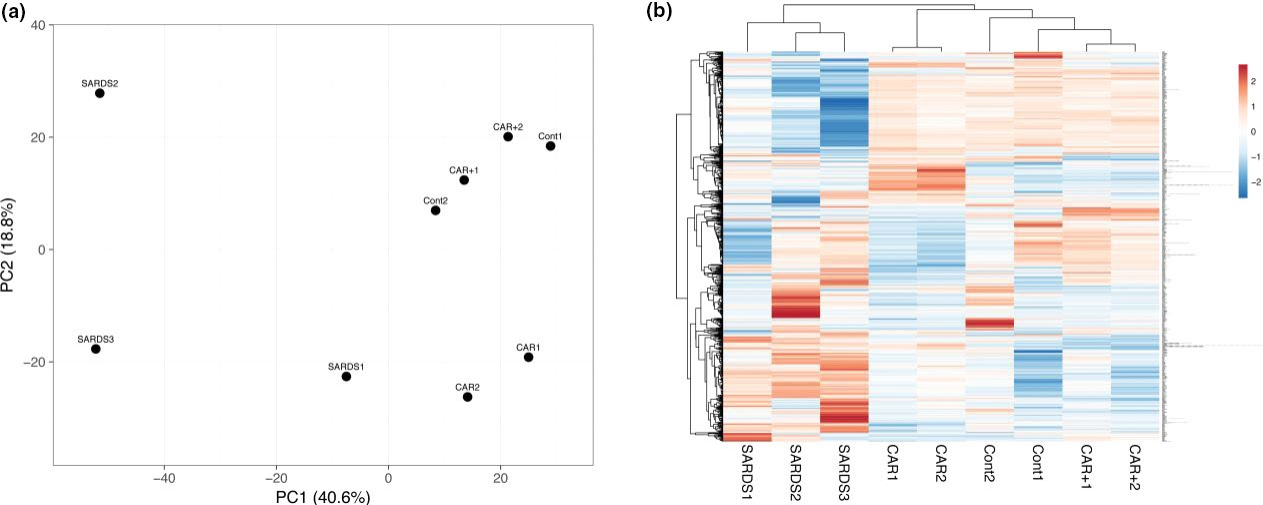
(A) PCA representing the 2500 genes with the highest variability in expression among the retinal samples used for transcriptional analysis. Principal component 1 (x‐axis) explains 40.6% of observed variation; principal component 2 (y‐axis) signifies 18.8% of variation. CAR1 and CAR2 are untreated CAR retinas from a same dog, CAR + 1 and CAR + 2 are CAR retinas following IVIg treatment from a same dog, SARDS1, SARDS2, and SARDS3 represent three SARDS eyes from three different dogs, and Cont1 and Cont2 are samples from normal healthy eyes of two healthy dogs. (B) Hierarchical cluster based on the 1200 genes with the most variable expression. Genes are clustered along the y‐axis. Each column represents a different sample. Red shades indicate higher expression, while blue shades signify lower expression

Gene expression analysis was carried out using retinal tissue from two eyes of one dog with untreated IMR‐CAR (CAR, patient 2, Table 1), two eyes from one IMR‐CAR dog who had received IVIg treatment (CAR+, patient 1, Table 1), two eyes from healthy control dogs (Control), and three eyes from three dogs with a diagnosis of SARDS (SARDS). After normalization and removal of probesets with low expression (log2 < 6) in all samples, 23 286 probesets remained for analysis. To evaluate the global relationship of the samples to one another, the 2500 probesets exhibiting the highest variability across all samples were identified and used for principal component analysis (PCA) while, due to the limitations of the software, the highest 1200 were used for hierarchical clustering. The results of the PCA (Figure 8A) demonstrate a close molecular relationship between the control samples and CAR+, concordant with the treatment success in these animals. CAR samples are located close together and near the controls, separated from them mostly due to differences in the lesser PC2 vector, indicating a relatively close relationship of the gene expression profiles. Retinal expression of the SARDS eye exhibited substantial variability between samples, but all were clearly distinct from both the CAR and control samples. These relationships were further confirmed using hierarchical clustering (Figure 8B). Here, the two tightest clusters were formed between the two CAR and CAR+ samples, which were then clustered with the control. Again, SARDS samples formed a distinct group, clustered separately from all other samples.

We then compared gene expression directly between control, CAR, and CAR+ samples (Tables 6, 7 and 8). Applying a signal cut‐off of log2 > 6 in at least one sample, statistical significance <0.05, and a twofold expression change identified 107 probesets representing 83 genes with elevated expression in the CAR retina, while 95 probesets representing 76 genes showed reduced expression in CAR. Functional annotation clustering of the genes indicated substantial increased transcription of immunoglobulins in CAR eyes. These transcripts were generally absent in control eyes but were expressed at a high level in the disease. We also detected increased expression of genes related to cancer signaling pathways, in particular those belonging to the PI3K‐Akt pathway, as well as a small number of chemokines. In addition, increased expression of metabolic pathways and ER or Golgi components might have been indicative of a heightened metabolic demand in these eyes. Notably absent in among these genes were those typically associated with inflammatory cells, suggesting that only a small number of leukocytes were present in the retinas, that is, those indicative of active inflammation of the tissue.

**Table 6:** Gene expression changes between control (cont) and CAR (CAR) retinas

Higher in CAR						
Probeset	Gene	Annotation	Cont	CAR	pVal	Fold Change
Immunoglobulin‐like domain						
Cfa.12195.14.S1_s_at*	LOC475754	Immunoglobulin gamma heavy‐chain B	5.04	12.76	0.003	211.08
Cfa.4465.2.S1_at	LOC608238	Ig lambda‐7 chain C region‐like	7.84	13.05	0.028	37.05
CfaAffx.21065.1.S1_s_at	LOC100687054	Immunoglobulin lambda‐like polypeptide 5	8.01	13.08	0.024	33.66
Cfa.4465.2.S1_s_at*	LOC486411	Similar to immunoglobulin lambda light chain	8.33	13.35	0.025	32.46
CfaAffx.23613.1.S1_x_at	LOC491492	Immunoglobulin V‐set domain	4.17	8.77	0.016	24.29
CfaAffx.23040.1.S1_x_at	LOC491391	Similar to immunoglobulin kappa light chain	3.79	7.36	0.029	11.90
CfaAffx.15259.1.S1_at*	ALCAM	Activated leukocyte cell adhesion molecule	7.30	10.19	0.003	7.38
CfaAffx.28261.1.S1_x_at	LOC490890	Ig heavy‐chain V region	4.70	7.42	0.039	6.62
CfaAffx.28271.1.S1_x_at	LOC607179	Ig heavy‐chain V region	5.27	7.65	0.045	5.21
Cfa.14528.1.A1_at**	DLA‐79	MHC class Ib	8.73	11.03	0.028	4.90
CfaAffx.20953.1.S1_x_at	LOC486374	Ig lambda chain V‐I region BL2	5.15	7.12	0.041	3.92
CfaAffx.539.1.S1_x_at	LOC490888	Ig heavy‐chain Mem5‐like	5.60	6.79	0.021	2.28
PI3K‐Akt, RAS signaling pathway/pathways in cancer						
Cfa.2937.1.A1_at*	PLA2G4A	Phospholipase A2 group IVA	7.10	10.57	0.016	11.09
CfaAffx.16027.1.S1_at	PRL	Prolactin	5.37	8.73	0.034	10.25
CfaAffx.10131.1.S1_s_at	KITLG	KIT ligand	5.85	7.89	0.016	4.12
Cfa.77.1.S1_s_at	PCK1	Phosphoenolpyruvate carboxykinase 1	4.82	6.54	0.032	3.30
CfaAffx.28421.1.S1_at	LAMA1	Laminin subunit alpha 1	4.48	5.88	0.017	2.64
Cfa.3581.1.S2_at	VEGFA	Vascular endothelial growth factor A	10.76	11.84	0.045	2.12
CfaAffx.7426.1.S1_s_at	PIK3R3	Phosphoinositide‐3‐kinase regulatory subunit 3	7.92	8.97	0.015	2.08
Chemokines						
CfaAffx.8579.1.S1_at	CXCR4	C‐X‐C motif chemokine receptor 4	5.31	7.52	0.008	4.65
Cfa.16327.1.S1_at*	CCR5	C‐C motif chemokine receptor 5	4.95	7.16	0.023	4.64
CfaAffx.5843.1.S1_s_at	ELMO1	Engulfment and cell motility 1	8.97	10.05	0.010	2.11
Negative Regulation of TGFb receptor signaling						
CfaAffx.19650.1.S1_at*	STRAP	Serine/threonine kinase receptor‐associated protein	8.52	10.50	0.010	3.95
Cfa.8414.1.A1_s_at	CIDEA	Cell death‐inducing DFFA‐like effector a	6.14	7.55	0.023	2.66
CfaAffx.22856.1.S1_s_at	SKIL	SKI‐like proto‐oncogene	5.43	6.60	0.032	2.25
ER/Golgi						
CfaAffx.18491.1.S1_s_at	ARV1	ARV1 homolog, fatty acid homeostasis modulator	5.74	8.79	0.000	8.28
CfaAffx.9366.1.S1_s_at*	BHLHE40	Basic helix‐loop‐helix family member e40	6.57	9.00	0.021	5.41
Cfa.18933.2.S1_s_at	CLEC2D	C‐type lectin domain family 2 member D	4.81	7.17	0.042	5.12
CfaAffx.27070.1.S1_at	SYT4	Synaptotagmin 4	5.52	6.86	0.045	2.53
CfaAffx.22494.1.S1_s_at**	P4HA1	Prolyl 4‐hydroxylase subunit alpha 1	7.82	8.95	0.024	2.19
Cfa.16398.1.S1_at	SULF2	Sulfatase 2	9.05	10.10	0.041	2.08
Metabolic Pathways						
Cfa.771.1.A1_at	ARG1	Arginase 1	6.75	7.95	0.027	2.30
Cfa.20672.1.S1_s_at	POLR2C	RNA polymerase II subunit C	7.48	8.51	0.008	2.05
CfaAffx.23757.1.S1_s_at	ALDH4A1	Aldehyde dehydrogenase 4 family member A1	5.89	6.90	0.009	2.01

Lower in CAR						
Epidermal growth factor‐like domain						
Cfa.14034.1.A1_at	LRP2	LDL receptor‐related protein 2	9.66	7.47	0.026	−4.56
CfaAffx.22339.1.S1_s_at*	IGFBP5	Insulin‐like growth factor‐binding protein 5	9.31	8.01	0.002	−2.45
Cfa.19619.1.A1_at	FBN2	Fibrillin 2	6.98	5.76	0.016	−2.34
Cfa.19661.1.S1_s_at	FBLN5	Fibulin 5	8.16	7.03	0.023	−2.20
CfaAffx.2075.1.S1_at	EGFL8	EGF‐like domain multiple 8	7.63	6.60	0.039	−2.04
PI3K‐Akt signaling pathway						
CfaAffx.3987.1.S1_s_at*	GNG11	G protein subunit gamma 11	10.41	8.96	0.006	−2.74
Cfa.496.1.A1_s_at	COL5A2	Collagen type V alpha 2 chain	8.80	7.30	0.047	−2.84
CfaAffx.13342.1.S1_at	VEGFC	Vascular endothelial growth factor C	8.05	6.67	0.045	−2.60
Other						
Cfa.260.1.S1_at	NOLC1	Nucleolar and coiled‐body phosphoprotein 1	12.70	9.05	0.001	−12.52
CfaAffx.6097.1.S1_at	VAX2	Ventral anterior homeobox 2	8.28	5.44	0.014	−7.13
Cfa.6923.1.A1_at	METTL3	Methyltransferase‐like 3	11.38	8.72	0.001	−6.30
Cfa.15672.1.A1_at*	TLCD1	TLC domain containing 1	9.92	7.71	0.010	−4.65
Cfa.1575.1.A1_s_at	MID2	Midline 2	6.41	4.30	0.019	−4.32
CfaAffx.26145.1.S1_s_at	RAD23A	RAD23 homolog A, nucleotide excision repair protein	9.63	7.56	0.001	−4.20
CfaAffx.20614.1.S1_s_at*	APOC3	Apolipoprotein C3	8.80	6.73	0.021	−4.19
CfaAffx.10606.1.S1_s_at	OXT	Oxytocin/neurophysin I	10.34	8.31	0.004	−4.10
Cfa.18689.1.S1_at**	A2M	Alpha‐2‐macroglobulin	8.14	6.15	0.026	−3.98
Cfa.14330.1.A1_at*	SLC22A6	Solute carrier family 22 member 6	9.63	7.68	0.020	−3.87
Cfa.20016.1.S1_at*	CFH	Complement factor H	9.04	7.15	0.003	−3.73
CfaAffx.7513.1.S1_at	RAB38	Member RAS oncogene family	8.12	6.23	0.018	−3.69
CfaAffx.28228.1.S1_at	ISL1	ISL1 transcription factor	8.72	6.89	0.002	−3.55
Cfa.21019.1.S1_at*	MAZ	MYC‐associated zinc finger protein	7.98	6.28	0.024	−3.27
Cfa.7069.1.A1_at	DDX3X	DEAD‐box helicase 3 X‐linked	8.94	7.26	0.001	−3.20
CfaAffx.25060.1.S1_at*	MPP5	Membrane palmitoylated protein 5	8.98	7.34	0.012	−3.10
CfaAffx.3688.1.S1_s_at	ABCC10	ATP binding cassette subfamily C member 10	8.66	7.03	0.002	−3.10
Cfa.17382.1.S1_s_at*	GPR177	Wnt ligand secretion mediator	7.72	6.19	0.035	−2.89
CfaAffx.13316.1.S1_at	SPON1	Spondin 1	9.31	7.82	0.049	−2.81
CfaAffx.14042.1.S1_at	VEPH1	Ventricular zone expressed PH domain containing 1	6.56	5.09	0.019	−2.77

Note

We show a portion of all detected changes along with functional classifications. Expression values represent log2 of detected signal strength. Note that one gene may be assigned to more than one functional category, but is only listed here once. One asterisk behind the probeset ID indicates that two probesets for the same gene were detected. Two asterisks indicate that at least three probesets were detected for this gene.

**Table 7:** Gene expression changes between CAR (CAR) and CAR + (IVIg‐treated CAR eye that responded to therapy) retinas

Higher in CAR						
Probeset	Gene	Annotation	CAR	CAR+	pVal	Fold Change
Immunoglobulin‐like domain						
Cfa.12195.14.S1_s_at*	LOC475754	Immunoglobulin gamma heavy‐chain B	12.76	5.08	0.004	205.56
Cfa.4465.2.S1_at	LOC608238	Ig lambda‐7 chain C region‐like	13.05	7.36	0.018	51.74
CfaAffx.21065.1.S1_s_at	LOC100687054	Immunoglobulin lambda‐like polypeptide 5	13.08	7.65	0.045	43.21
Cfa.4465.2.S1_s_at	LOC486411	Similar to immunoglobulin lambda light chain	13.35	7.95	0.045	42.30
CfaAffx.23613.1.S1_x_at	LOC491492	Immunoglobulin V‐set domain	8.77	4.37	0.017	21.15
CfaAffx.23040.1.S1_x_at	LOC491391	Similar to immunoglobulin kappa light chain	7.36	3.94	0.032	10.72
CfaAffx.28261.1.S1_x_at	LOC490890	Ig heavy‐chain V region	7.42	4.79	0.041	6.20
CfaAffx.28271.1.S1_x_at	LOC607179	Ig heavy‐chain V region	7.65	5.35	0.044	4.94
Cfa.14528.1.A1_at**	DLA‐79	MHC class Ib	11.03	8.72	0.005	4.94
CfaAffx.20953.1.S1_x_at	LOC486374	Ig lambda chain V‐I region BL2	7.12	5.21	0.038	3.76
CfaAffx.539.1.S1_x_at	LOC490888	Ig heavy‐chain Mem5‐like	6.79	5.70	0.032	2.12
PI3K Pathways						
CfaAffx.21308.1.S1_s_at*	PLA2G4A	Phospholipase A2 group IVA	9.62	5.40	0.009	18.63
Cfa.1213.1.S1_s_at*	NR4A1	Nuclear receptor subfamily 4 group A member 1	11.70	7.75	0.002	15.48
Cfa.3528.1.S1_s_at	IL‐6	Interleukin 6	7.90	4.79	0.017	8.59
Cfa.77.1.S1_s_at	PCK1	Phosphoenolpyruvate carboxykinase 1	6.54	4.64	0.003	3.73
CfaAffx.22274.1.S1_x_at*	DDIT4	DNA damage‐inducible transcript 4	8.94	7.41	0.004	2.88
Cfa.1262.1.S1_s_at	COL1A2	Collagen type I alpha 2 chain	7.38	5.91	0.026	2.77
CfaAffx.30925.1.S1_s_at	PHLPPL	PH domain and leucine‐rich repeat protein phosphatase 2	5.96	4.76	0.025	2.29
Cfa.3581.1.S2_at*	VEGFA	Vascular endothelial growth factor A	11.84	10.80	0.006	2.05
AP‐1 transcription factor						
CfaAffx.26065.1.S1_at*	FOS	Fos proto‐oncogene, AP‐1 transcription factor subunit	10.98	7.05	0.002	15.23
CfaAffx.26260.1.S1_at	JUNB	JunB proto‐oncogene, AP‐1 transcription factor subunit	9.15	6.60	0.002	5.86
CfaAffx.6.1.S1_s_at	FOSB	FosB proto‐oncogene, AP‐1 transcription factor subunit	6.73	5.02	0.003	3.27
CfaAffx.8766.1.S1_at	FOSL2	FOS‐like 2, AP‐1 transcription factor subunit	6.79	5.72	0.003	2.11
Negative Regulation of TGFb receptor signaling						
Cfa.8414.1.A1_s_at	CIDEA	Cell death‐inducing DFFA‐like effector a	7.55	4.63	0.005	7.58
CfaAffx.19650.1.S1_at*	STRAP	Serine/threonine kinase receptor‐associated protein	10.09	7.94	0.038	4.45
ER/Golgi						
CfaAffx.18491.1.S1_s_at*	ARV1	ARV1 homolog, fatty acid homeostasis modulator	8.79	5.86	0.000	7.62
CfaAffx.9366.1.S1_s_at**	BHLHE40	Basic helix‐loop‐helix family member e40	9.00	6.10	0.017	7.46
Cfa.18933.2.S1_s_at	CLEC2D	C‐type lectin domain family 2 member D	7.17	4.46	0.023	6.56
Cfa.14057.1.A1_at	ACSL3	Acyl‐CoA synthetase long‐chain family member 3	6.71	4.38	0.010	5.04
Cfa.13172.1.S1_at*	HSD3B2	Hydroxy‐delta‐5‐steroid dehydrogenase, 3 beta‐ and steroid delta‐isomerase 2	10.31	8.36	0.000	3.88
Cfa.10767.1.S1_s_at	CYP3A12	Cytochrome P‐450 3A12	8.31	6.45	0.033	3.62
CfaAffx.21662.1.S1_at	SAR1A	Secretion‐associated Ras‐related GTPase 1A	7.39	6.11	0.018	2.43
Cfa.11838.1.A1_s_at*	TMX4	Thioredoxin‐related transmembrane protein 4	7.99	6.73	0.026	2.39
CfaAffx.22817.1.S1_at	GNPNAT1	Glucosamine‐phosphate N‐acetyltransferase 1	7.74	6.55	0.044	2.28
Metabolic Pathways						
Cfa.2937.1.A1_at	PLA2G4A	Phospholipase A2 group IVA	10.57	6.43	0.011	17.64
CfaAffx.3596.1.S1_s_at**	ALDH1A1	Aldehyde dehydrogenase 1 family member A1	12.03	8.77	0.028	9.55
Cfa.2170.1.S1_at	ALDH1B1	Aldehyde dehydrogenase 1 family member B1	9.16	6.25	0.009	7.53
CfaAffx.15778.1.S1_at	CMBL	Carboxymethylenebutenolidase homolog	8.68	6.92	0.010	3.38
Cfa.19154.1.S1_a_at*	CMPK2	Cytidine/uridine monophosphate kinase 2	6.33	5.26	0.026	2.11
CfaAffx.21433.1.S1_at	RIMKLB	Ribosomal modification protein rimK‐like family member B	7.19	6.15	0.004	2.06
Cfa.15684.1.A1_s_at	POLR3F	RNA polymerase III subunit	8.99	7.98	0.006	2.01

Lower in CAR						
TGFb pathways						
CfaAffx.22914.1.S1_at	BMP4	Bone morphogenetic protein 4	7.72	9.42	0.027	−3.24
Cfa.7153.1.A1_s_at*	BMP2	Bone morphogenetic protein 2	9.03	10.52	0.007	−2.82
CfaAffx.3587.1.S1_at	BTBD11	BTB domain containing 11	5.92	7.24	0.009	−2.50
CfaAffx.18662.1.S1_at	BMP7	Bone morphogenetic protein 7	8.06	9.17	0.007	−2.15
CfaAffx.1077.1.S1_at	GDF11	Growth differentiation factor 11	8.23	9.24	0.014	−2.01
FAD Synthesis and FAD dependent enzymes						
Cfa.12126.1.A1_at*	GCDH	Glutaryl‐CoA dehydrogenase	6.83	9.77	0.004	−7.67
Cfa.12618.1.A1_at	DDO	D‐aspartate oxidase	5.36	7.97	0.011	−6.08
CfaAffx.14436.1.S1_at	DMGDH	Dimethylglycine dehydrogenase	5.21	6.82	0.026	−3.05
Cfa.12167.1.A1_at	XDH	Xanthine dehydrogenase	5.45	6.96	0.013	−2.84
Cfa.15688.1.A1_at*	DAO	D‐amino acid oxidase	5.00	6.38	0.003	−2.60
CfaAffx.11088.1.S1_at	CYB5R2	Cytochrome b5 reductase 2	6.35	7.64	0.040	−2.45
CfaAffx.21188.1.S1_s_at	DUOX1	Dual oxidase 1	5.03	6.08	0.030	−2.07
Innate Immune Response						
Cfa.7069.1.A1_at	DDX3X	DEAD‐box helicase 3, X‐linked	7.26	9.87	0.004	−6.10
CfaAffx.26852.1.S1_at	S100A8	S100 calcium‐binding protein A8	7.10	9.48	0.003	−5.22
CfaAffx.30748.1.S1_at	LCN2	Lipocalin 2	5.92	8.38	0.033	−5.50
Cfa.6458.2.A1_at	CFB	Complement factor B	7.61	9.49	0.009	−3.67
Cfa.20016.1.S1_at*	CFH	Complement factor H	7.15	9.62	0.004	−5.54
Intermediate filament						
CfaAffx.438.1.S1_s_at	KRT18	Keratin 18	9.23	11.43	0.008	−4.59
Cfa.7839.1.S1_at**	KRT8	Keratin 8	7.85	9.37	0.005	−2.86
CfaAffx.24552.1.S1_at	KRT12	Keratin 12	5.65	6.82	0.038	−2.26
CfaAffx.24556.1.S1_s_at	KRT10	Keratin 10	8.73	9.87	0.005	−2.20
PI3K‐Akt signaling pathway						
Cfa.3626.1.A1_s_at	FGFR2	Fibroblast growth factor receptor 2	8.82	10.92	0.040	−4.28
Cfa.10634.1.A1_at*	ITGA7	Integrin subunit alpha 7	9.54	10.82	0.005	−2.43
Cfa.14.1.A1_at*	LAMA3	Laminin subunit alpha 3	8.34	9.61	0.000	−2.42
CfaAffx.14209.1.S1_s_at	THBS4	Thrombospondin 4	6.01	7.42	0.009	−2.66
CfaAffx.13342.1.S1_at	VEGFC	Vascular endothelial growth factor C	6.67	8.02	0.028	−2.56
Lipid Metabolism						
CfaAffx.20614.1.S1_s_at*	APOC3	Apolipoprotein C3	6.73	9.57	0.010	−7.13
CfaAffx.24089.1.S1_at	LIPA	Lipase A, lysosomal acid type	6.23	8.38	0.039	−4.45
Cfa.12190.1.A1_at	PTGES	Prostaglandin E synthase	9.13	10.40	0.023	−2.41
Cfa.19665.1.A1_at	PLCL1	Phospholipase C‐like 1	8.51	9.65	0.003	−2.20
Melanogenesis						
CfaAffx.1094.1.S1_at	SILV	Premelanosome protein	8.02	12.14	0.001	−17.42
CfaAffx.7483.1.S1_s_at	TYR	Tyrosinase	5.58	9.64	0.006	−16.75
Cfa.193.1.A1_at	TYRP1	Tyrosinase‐related protein 1	5.03	8.92	0.009	−14.88
CfaAffx.8921.1.S1_at	DCT	Dopachrome tautomerase	7.40	10.69	0.019	−9.79
Cfa.4886.1.A1_at	OCA2	Oculocutaneous albinism 2	6.28	9.23	0.010	−7.72

Note

A portion of all detected changes are shown along with functional classifications. Expression values represent log2 of detected signal strength. Note that one gene may be assigned to more than one functional category, but is listed only once here. One asterisk behind the probeset ID indicates that two probesets for the same gene were detected. Two asterisks indicate that at least three probesets were detected for this gene.

**Table 8:** Gene expression changes between CAR (CAR) and SARDS (SARDS) retinas

Higher in CAR						
Probeset	Gene	Annotation	CAR	SARDS	pVal	Fold Change
Immunoglobulin Related						
Cfa.12195.14.S1_s_at*	LOC475754	Immunoglobulin gamma heavy‐chain B	12.76	8.15	0.003	24.49
CfaAffx.23613.1.S1_x_at	LOC491492	Similar to immunoglobulin kappa light chain	8.77	4.74	0.005	16.29
CfaAffx.23040.1.S1_x_at	LOC491391	Similar to immunoglobulin kappa light chain	7.36	3.76	0.005	12.17
CfaAffx.28261.1.S1_x_at	LOC490890	Ig heavy‐chain Mem5‐like	7.42	4.86	0.021	5.92
CfaAffx.28271.1.S1_x_at	LOC607179	Ig heavy‐chain V region	7.65	5.88	0.026	3.42
CfaAffx.539.1.S1_x_at	LOC490888	Ig heavy‐chain Mem5‐like	6.79	5.76	0.013	2.04
MAPK signaling pathway						
Cfa.1213.1.S1_s_at*	NR4A1	Nuclear receptor subfamily 4 group A member 1	11.70	7.28	0.003	21.42
CfaAffx.26065.1.S1_at*	FOS	Fos proto‐oncogene, AP‐1 transcription factor subunit	10.98	8.52	0.006	5.52
Cfa.2667.1.S1_s_at	CACNB2	Calcium voltage‐gated channel subunit beta 2	8.10	6.33	0.037	3.41
CfaAffx.25714.1.S1_at*	DUSP1	Dual specificity phosphatase 1	8.05	6.49	0.004	2.94
Cfa.17755.1.S1_s_at*	PPP3CC	Protein phosphatase 3 subunit gamma	10.24	8.81	0.027	2.70
PI3K‐Akt signaling pathway						
Cfa.3528.1.S1_s_at	IL‐6	Interleukin 6	7.90	5.35	0.010	5.86
Cfa.77.1.S1_s_at	PCK1	Phosphoenolpyruvate carboxykinase 1	6.54	4.90	0.018	3.14
CfaAffx.22274.1.S1_x_at*	DDIT4	DNA damage‐inducible transcript 4	8.94	7.34	0.001	3.02
CfaAffx.30925.1.S1_s_at*	PHLPPL	PH domain and leucine‐rich repeat protein phosphatase 2	5.96	4.36	0.024	3.02
Cfa.3581.1.S1_s_at*	VEGFA	Vascular endothelial growth factor A	7.62	6.29	0.004	2.52
Metabolic pathways						
Cfa.18560.1.S1_s_at	TKTL1	Transketolase‐like 1	9.55	5.74	0.037	14.01
Cfa.825.1.S1_s_at**	SLC2A3	Glucose transporter type 3, brain	8.47	5.28	0.001	9.16
CfaAffx.12528.1.S1_at	GALNTL6	Polypeptide N‐acetylgalactosaminyltransferase‐like 6	7.24	5.31	0.002	3.82
Cfa.18746.1.S1_s_at	PPAP2C	Phospholipid phosphatase 2	11.22	9.51	0.047	3.26
Cfa.13313.2.S1_at	INPP1	Inositol polyphosphate‐1‐phosphatase	7.65	5.95	0.014	3.24
CfaAffx.11455.1.S1_at	EHHADH	Enoyl‐CoA hydratase and 3‐hydroxyacyl CoA dehydrogenase	10.79	9.13	0.042	3.15
Cfa.13804.1.A1_at	ALPL	Alkaline phosphatase	6.44	5.02	0.038	2.67
CfaAffx.28588.1.S1_at	PIGS	Phosphatidylinositol glycan anchor biosynthesis class S	8.68	7.34	0.008	2.52
Cfa.15397.1.A1_at	MCCC2	Methylcrotonyl‐CoA carboxylase 2	7.74	6.42	0.000	2.50
Cfa.19793.1.S1_at	LOC477508	Cytochrome c oxidase subunit 6A1, mitochondrial	7.69	6.38	0.005	2.48
Cfa.18326.1.S1_s_at*	SORD	Sorbitol dehydrogenase	7.25	6.04	0.009	2.31
CfaAffx.2615.1.S1_s_at	ST3GAL1	ST3 beta‐galactoside alpha‐2,3‐sialyltransferase 1	5.96	4.79	0.001	2.25
CfaAffx.18122.1.S1_at	ETNK1	Ethanolamine kinase 1	7.62	6.55	0.039	2.11
Cfa.15684.1.A1_s_at	POLR3F	RNA polymerase III subunit F	8.99	7.94	0.018	2.06
Cancer associated						
CfaAffx.20006.1.S1_at**	CDC2	Cyclin‐dependent Kinase 2	11.26	7.19	0.041	16.84
CfaAffx.19651.1.S1_s_at*	STRAP	Serine/threonine kinase receptor‐associated protein	10.09	8.51	0.004	3.00
Cfa.9822.1.S1_s_at	ABCG2	Breast cancer resistance protein 1	9.31	7.78	0.019	2.90
Cfa.12569.1.A1_at	LYAR	Ly1 antibody reactive	11.24	9.86	0.044	2.59
CfaAffx.16126.1.S1_s_at	ABCG1	ATP binding cassette subfamily G member 1	6.80	5.53	0.020	2.41
CfaAffx.13657.1.S1_s_at	CCDC158	Coiled‐coil domain containing 158	7.54	6.48	0.020	2.08

Higher in SARDS						
Proinflammatory Signaling/neutrophil chemotaxis						
Cfa.16590.1.S1_s_at*	CXCL10	Chemokine (C‐X‐C motif) ligand 10	6.77	8.78	0.017	4.03
Cfa.40.1.S1_s_at	IL‐18	Interleukin 18	6.43	7.93	0.046	2.23
CfaAffx.24815.1.S1_at	TLR1	Toll‐like receptor 1	6.81	8.20	0.007	1.93
CfaAffx.22164.1.S1_s_at	LGALS3	Galectin 3	9.35	10.62	0.049	1.60
Cfa.12237.1.A1_at	CCL23	Chemokine (C‐C motif) ligand 23	10.68	11.86	0.039	1.39
Cfa.3634.1.S1_at	ITGB2	Integrin subunit beta 2	7.15	8.30	0.017	1.32
Cfa.6225.1.A1_at	TNFRSF14	TNF receptor superfamily member 14	7.35	8.45	0.041	1.21
CfaAffx.6888.1.S1_at	MAP3K8	Mitogen‐activated protein kinase kinase kinase 8	5.25	6.34	0.004	1.19
CfaAffx.21295.1.S1_at	CCRL2	C‐C motif chemokine receptor‐like 2	6.15	7.19	0.029	1.07
T/B/NK cell markers						
Cfa.14560.1.S1_at	CD48	Leukocyte antigen MEM‐102	7.88	9.33	0.046	2.09
CfaAffx.30242.1.S1_at	CD53	Leukocyte surface antigen CD53	7.03	8.26	0.012	1.50
Cfa.3629.2.S1_s_at	CD86	B‐Lymphocyte antigen B7‐2	8.82	10.01	0.025	1.42
CfaAffx.19610.1.S1_at	IFGGB1	T cell–specific guanine nucleotide triphosphate‐binding protein 1	4.94	6.08	0.021	1.30
Cfa.3596.2.S1_s_at	CD80	T‐lymphocyte activation antigen CD80	6.40	7.43	0.043	1.07
Cfa.21258.1.S1_at	FCGR3A	CD16a antigen	7.94	8.95	0.037	1.02
Antigen presentation						
Cfa.182.1.S2_at*	DLA‐DQA1	Major histocompatibility complex, class II, DQ alpha 1	8.72	10.39	0.015	2.79
CfaAffx.2152.1.S1_s_at	DLA‐DQB1	HLA class II histocompatibility antigen, DQ beta 2 chain‐like	9.09	10.42	0.014	1.77
CfaAffx.2126.1.S1_s_at	DLA‐DRA1	MHC class II DR alpha chain	10.98	12.09	0.025	1.24
Cfa.12298.1.A1_a_at*	PSMB8	Proteasome subunit beta 8	9.11	10.20	0.010	1.17
Cfa.1333.3.S1_a_at	CD74	CD74 molecule	11.79	12.83	0.002	1.08
Negative Regulation of Inflammation						
Cfa.18689.1.S1_s_at**	A2M	Alpha‐2‐macroglobulin	6.62	10.25	0.012	13.18
CfaAffx.12376.1.S1_at**	CNTF	Ciliary neurotrophic factor	6.19	8.01	0.003	3.30
Cfa.9474.1.A1_s_at	CLEC12A	C‐type lectin domain family 12 member A(CLEC12A)	8.73	10.17	0.013	2.07
Cfa.3452.1.S1_s_at	PTGER4	Prostaglandin E receptor 4	6.90	8.32	0.017	2.03
Cfa.12136.1.A1_at	ADA	Adenosine deaminase	6.07	7.49	0.008	2.01
Cfa.16454.1.S1_at*	BTK	Bruton tyrosine kinase	6.23	7.63	0.032	1.95
CfaAffx.8336.1.S1_at	PLCD1	Phospholipase C delta 1	5.70	6.89	0.002	1.40
Cfa.12235.1.A1_s_at	TYROBP	TYRO protein tyrosine kinase binding protein(TYROBP)	9.51	10.67	0.027	1.33
Cfa.3166.1.S1_at	TYRO3	TYRO3 protein tyrosine kinase	7.38	8.40	0.042	1.03

Note

Expression values represent log2 of detected signal strength. We show a portion of all detected changes along with functional classifications. Expression values represent log2 of detected signal strength. Note that one gene may be assigned to more than one functional category, but is listed only once. One asterisk behind a probeset ID indicates that two probesets for the same gene were detected. Two asterisks indicate that at least three probesets were detected for this gene.

Reduced expression was observed for a largely disparate group of genes, although expression changes were typically mild. However, decreased expression of a number of genes containing an epidermal growth factor (EGF) domain was noted, as were molecules limiting PI3K‐Akt pathway signaling.

When the gene expression patterns of the CAR samples were compared to those from SARDS eyes, we detected 254 probesets representing 199 genes with a higher expression in CAR, and 167 probesets of 136 genes more prominently expressed in SARDS retinas. As noted above, CAR samples expressed a substantial amount of immunoglobulins. As in our previous study, mRNA encoding immunoglobulins was also present in SARDS eyes, but to a significantly smaller extent than in CAR.^5^ Additionally, we observed increased transcripts related to mitogen‐activated protein kinase (MAPK) and PI3K‐Akt signaling, and elevated expression of a number of genes that have been associated with the development of cancer. Finally, a number of transcriptional changes indicated that CAR samples had increased metabolic demand.

In contrast, SARDS eyes displayed significantly higher expression of proinflammatory molecules, including IL‐18, TLR1, and chemokines and their receptors (Table 8). We also detected higher transcriptional activity for gene products related to antigen presentation, primarily related to MHC class II. Finally, significantly higher expression of genes associated with T, B, or NK cells was observed in the SARDS samples compared to CAR. The presence of these transcripts most likely indicates that a larger number of these cells infiltrate into the SARDS retina than the CAR.

### 3.9 Treatment outcomes in canine CAR‐IMR patients

**Figure 9:**
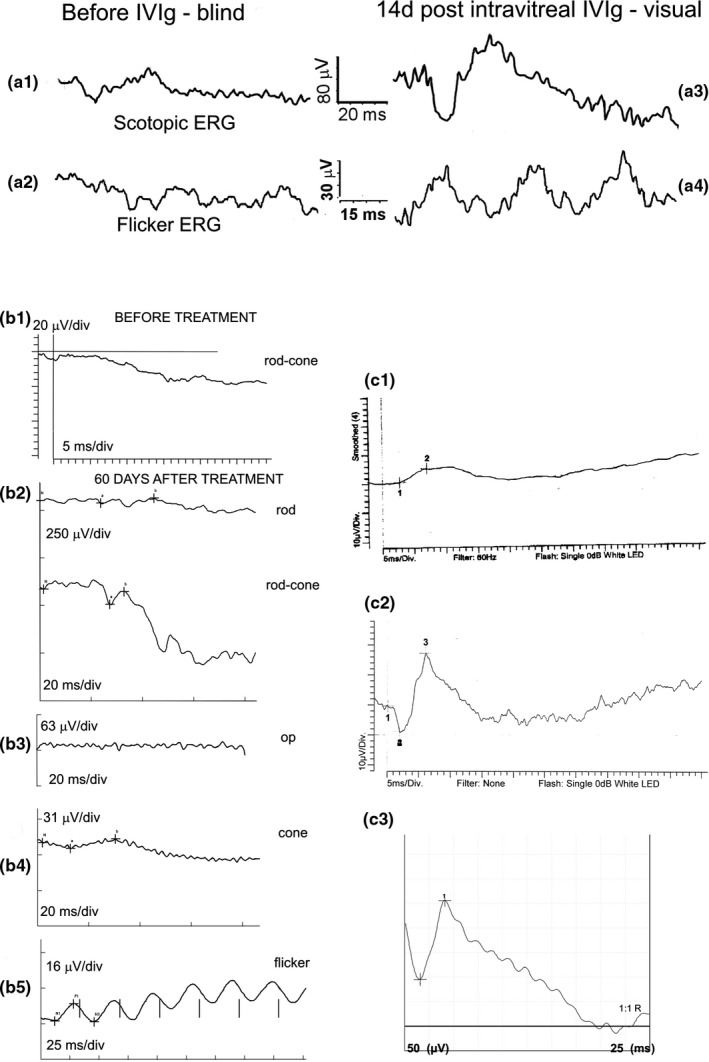
ERG tracings in CAR‐treated canine patients. (A1‐A2) Patient 1, Table 1, meningioma: ERG testing revealed severe decrease in full‐field scotopic ERG amplitudes and flicker ERG amplitudes with development of blindness despite very high dose of systemic steroids. Complete recovery of vision in photopic conditions with significant improvement in retinal electrical activity was detected 14 d after intraocular IVIg injection. Dog remained blind in scotopic conditions. (B1‐B5) ERG tracings from the CAR patient (Patient 16, Table 1) with sarcoma before and after intravitreal IVIg therapy: (B1) Full‐field ERG tracings showed completely absent retinal electrical activity in both eyes (the pre‐treatment recording was done by primary ophthalmologist using the Retinographics full‐field combined rod‐cone routine; no other ERG testing routines were pursued). Patient was completely blind at presentation despite treatment with immunosuppressive dose of systemic steroids; (B2) rod response and full‐field scotopic combined rod‐cone response showed mild recovery in retinal electrical function 60 d after intravitreal IVIg injection; (B3) oscillatory potentials (op) were not recordable at the same time point; (B4) photopic cone and flicker response (B5) recordings showed recovery of cone electrical activity. Patient remained visual in photopic and mesopic conditions for 12 mo post‐treatment. (C1‐C3) ERG tracings from the CAR patient (Patient 3, Table 1) with meningioma: (C1) Full‐field ERG tracings showed by primary ophthalmologist revealed dramatic reduction in ERG amplitudes (b‐ wave = 22 µV) and absent cPLR after red light illumination with mildly decreased but present cPLR after the blue light illumination. Systemic steroids were initiated. (C2) Retinal electrical evaluation performed 7 d after the initiation of steroid therapy revealed normalization of responses (b‐wave amplitude = 130 µV, recordings performed at our institution). Vision was significantly improved. Twelve months after initiating treatment, sudden onset of complete vision loss was detected and ERG evaluation revealed 33 µV b‐wave amplitudes (ERG tracings not available, performed by primary ophthalmologist), and patient was referred to us for intravitreal IVIg treatment resulting in significant vision improvement (object tracking capability in day light conditions was restored). (C3) Completely normalized retinal electrical activity is present 22 mo after IVIg treatment; however, owner reports development of intermittent episodes of decreased vision, which correspond to episodes of pancreatitis flare ups. Due to the pancreatitis episodes and dramatic liver enzyme elevations, systemic steroids were first substituted with systemic cyclosporine (5 mg/kg BW q24h PO), which was not tolerated. Systemic cyclosporine was substituted with leflunomide (4 mg/kg BW q24h PO), which was tolerated for the period of 6 mo, and had to be discontinued. At that time, therapy had to be continued with subconjunctival steroid injections (monthly) and intravitreal steroid injections (once every 6 mo) combined with the low dose of systemic steroids (0.5 mg/kg BW q48h PO). Only full‐field combined rod‐cone routines were done for this patient during all examination time points when ERG testing was pursued. Legend: BW—body weight

**Figure 10:**
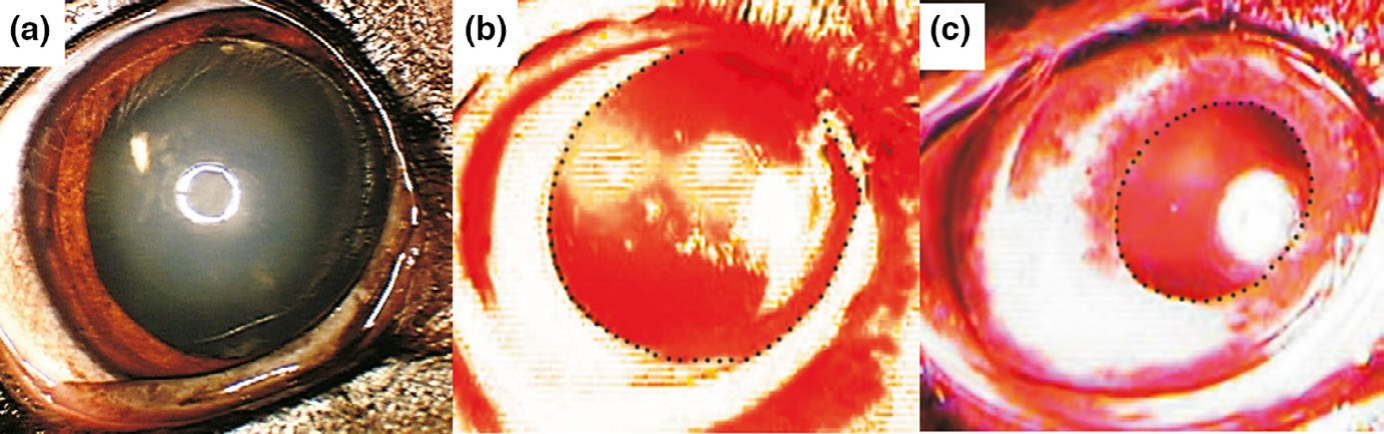
cPLR testing in CAR patient before and after intraocular IVIg treatment (Patient 1, Table 1, meningioma). (A) Resting pupil diameter in photopic conditions before IVIg treatment; (B) red light illumination did not elicit pupil light reflex before IVIg treatment; (C) recovery of the PLR after red light illumination was detectable 14 d after intravitreal IVIg injection

Immunosuppressive therapy resulted in the reversal of complete blindness in 44% (4 of 9) of treated patients, with 61% of all treated patients recovering and/or maintaining vision (8 of 13, Table 3). Median time for dogs maintaining vision was five months (range 1‐35 months), which corresponded to survival time. Initially, 53% (9 of 17) patients were treated with high doses of systemic steroids (prednisone: n = 6; prednisone + doxycycline: n = 3), with only 22% (2 of 9) responding to therapy, while 78% (7 of 9) did not respond. A total of four nonresponding patients were treated with the intravitreal IVIg injection, and three of those (75%) responded with the recovery of vision and improvement of cPLR and retinal electrical responses (Figures 9 and 10).

## 4 DISCUSSION

Human AIR (npAIR and CAR) are relatively rare autoimmune forms of diseases characterized by progressive loss of vision, severe depression of retinal electrical activity, minimal fundus changes, and presence of retinal autoAbs in the serum samples of human patients.^27^, ^28^ CAR was first reported in human patients almost five decades ago; however, reports of the CAR in the veterinary patient population have not been previously published in the peer‐reviewed literature.^41^ Grozdanic et al (2008) introduced a new classification of the possible AIR in dogs, proposing a classification separating a nonparaneoplastic clinical entity (SARDS), and IMR, in which some patients fit the criteria of CAR, while the majority did not have evidence of cancer.^2^

Sudden acquired retinal degeneration syndrome was first reported almost four decades ago, and in recent years, there has been sufficient evidence characterizing it as an immune‐mediated type of retinopathy.^5^, ^6^ Blindness caused by SARDS is most frequently seen in middle‐aged/older females of small breeds, with mixed‐breed dogs, Dachshunds, Pugs, and Miniature Schnauzers being the most frequently affected.^42^ In this study, there was an equal distribution between the large and small breed dogs affected, with males being more prevalent compared to females.

Sudden acquired retinal degeneration syndrome diagnostic features have been previously described: sudden onset of blindness, completely extinguished retinal electrical responses, absent chromatic PLR after red light illumination, normal chromatic PLR after the blue light illumination, and near‐normal fundus appearance.^2^, ^5^, ^34^ In this study, none of the patients evaluated satisfied previously established criteria for SARDS diagnosis, and we have tried to describe critical clinical features which may help differentiating CAR and SARDS patients (Table 9). While seven had completely extinguished full‐field electrical responses when combined rod‐cone evaluation ERG routine was used, none had the characteristic chromatic PLR deficits previously described in SARDS patients (no red‐good blue response). We detected a variety of retinal electrical responses in rest of the patients, which again is not a feature previously reported in SARDS patients. Observed combination of cPLR and electrophysiological changes in this patient population corresponded to the features previously described in IMR patients.^2^

**Table 9:** Proposed classification of autoimmune retinopathies (AIR) in canines based on the clinical presentation and currently established human nomenclature

	Autoimmune retinopathy (AIR) in canines^2^					
	CAR	Nonparaneoplastic autoimmune retinopathy (npAIR)				
	IMR^2^			SARDS		
	IMR‐CAR^2^, ^54^	npIMR^2^	Early IMR^55^	Early SARDS^55^	Intermediate SARDS^56^	Advanced SARDS^2^
Vision	Blind (usually)	Blind	Nyctalopia or normal	Nyctalopia or normal	Nyctalopia or normal	Blind
cPLR	−R, +B ±R, +B +R/+B −R/−B	−R, +B ±R, +B +R/+B −R/−B	−R, +B ±R, +B +R/+B −R/−B	−R, +B	−R, +B	−R, +B
ERG	Absent or decreased	Absent or decreased	Normal or decreased	Decreased but present	Absent	Absent
Response to therapy (%)	60%	35%‐50%	95%	95%	80%	35%‐40%
Therapy	Mono‐ or Bitherapy ± IVIg ± triamcinolone (io)	Bitherapy + IVIg + triamcinolone (io)*	Monotherapy	Monotherapy	Bitherapy + triamcinolone (subconj) ± IVIg (io)	Bitherapy + IVIg + triamcinolone (io)*

Note

IMR—immune‐mediated retinitis—terminology introduced in 2008 to differentiate patients with detectable ERG response or detectable chromatic pupil light reflex (cPLR) responses after the red light illumination when compared to sudden acquired retinal degeneration syndrome (SARDS) patients.

io, intravitreal IVIg application.

* Personal communication (pertinent to intravitreal IVIg administration)

Detailed fundus evaluation revealed numerous funduscopic abnormalities, very similar to those observed in SARDS eyes and described in a recent paper. Retinal vascular attenuation was the most frequently observed.^5^ Presence of frequently observed retinal inflammatory lesions such as perivascular hyper‐reflective lesions, perivascular retinal edema, and perivascular exudative lesions potentially support the notion of the possible inflammatory nature of the disease, and identical findings have been recently described in SARDS retinas.^5^

This study effectively utilized SD‐OCT technology to demonstrate presence of many retinal lesions (loss of the inner and outer segment photoreceptor stratification, loss of the outer segments, loss of the outer nuclear layer, retinal detachment, focal retinal thinning, and chorioretinal scars) previously reported in SARDS eyes, which were not easily detected by indirect ophthalmoscopy fundus evaluation or that of retinal fundus images.^5^ Careful analysis of retinal OCT scans revealed predominantly perivascular localization of lesions with inflammatory appearance, which was also confirmed by histopathological analysis of limited retinal tissue specimens.

An equally important finding of this study was the existence of the loss of the inner segment‐outer segment (IS‐OS) photoreceptor delineation, and presence of photoreceptor loss, which was particularly prominent in perivascular regions and frequently had a focal appearance. Identical findings have been previously described as the most frequently observed features in human npAIR and CAR patients.^43^, ^44^ Considering that all patients evaluated in this study had received a diagnosis of cancer, and many functional, structural, and funduscopic features had been previously observed in human CAR patients, we hypothesized that observed changes are indeed a result of the paraneoplastic retinal syndrome. However, we cannot exclude the possibility that these patients had an immune‐mediated form of retinal disease independent of the concurrent cancer presence.

In previous publications, it has been demonstrated histological evidence of perivascular T‐ and B‐cell inflammatory exudates, and MA evidence of inflammatory gene profile changes and primary photoreceptor lesions in perivascular regions observed by OCT imaging in SARDS retinas.^2^, ^5^ Similar findings were confirmed in this study to some extent, further reinforcing the notion of possible immune‐mediated events being responsible for observed functional and structural retinal changes. This is further supported by the fact that systemic and intraocular immunosuppressive and immunomodulatory therapy resulted in the restoration and preservation of vision in the majority of treated patients, as is frequently case in human CAR.^45^

Previous studies found a relatively high incidence of metabolic abnormalities in SARDS patients, such as PU and PD (38%,^42^ 30%^1^), PP (19%,^42^ 20%^1^), and SAP elevation (37%,^46^ 28%^1^), which were in good accord with our findings in this study. This potentially indicates a very similar nature of systemic changes when comparing SARDS and CAR patients. This study confirmed very high incidence of proteinuria (75%), which was similar to the recent data from SARDS patients in Canada, but much higher than previously published studies from the SARDS patient population in the United States.^5^, ^46^, ^47^

In this study, we attempted to characterize molecular events associated with CAR retinal damage in dogs, with the goal of better understanding the pattern of observed morphological and functional changes. This report is the first to describe detailed changes in the gene expression pattern and potential immunological consequences in species with large eyes, and spontaneously occurring disease similar to CAR in humans. A major limitation of the study was the small number of CAR samples analyzed. While this likely influenced the expression levels of some genes, the overall similarity between immunoglobulin gene expression in this study and our previously reported data from SARDS retina suggest that observed gene expression changes may indeed reflect similarities in the immunological reaction, with SARDS immunological profile being more aggressive compared to CAR.^5^ As a consequence of the small sample size, expression changes of individual genes should be regarded with care but more confidence can be placed in the identified pathways as these are supported by multiple genes. Furthermore, while it is conceivable that the eyes used for gene expression analysis represented atypical cases, this concern is alleviated by the overall similarity between immunoglobulin gene expression in this study and our previously reported SARDS data.^5^ Interestingly, molecular profiling and gene expression map comparison between SARDS and CAR eyes showed distinct differences in overall gene expression despite almost identical clinical presentation in many cases; however, difference in therapeutic response (60% in this study compared to 35%‐40% for blind SARDS patients, Table 9) should serve as an additional evidence of the more aggressive nature of disease in SARDS patients.

Among genes with elevated expression levels in CAR retinas, genes mediating various aspects of immune‐mediated response were by far the most prevalent. Prominent functional categories of genes with elevated expression in CAR retinas included antigen presentation, leukocyte activation and adhesion, lysosomal and proteasome activity, and immunoglobulin production. In addition, numerous genes with a function in apoptosis and inflammation signaling were more abundant in CAR retinas. Our analyses also indicated that CAR led to lower expression levels for a large number of genes, primarily associated with photoreceptor function. Reduced mRNA levels of individual genes could partially be the result of transcriptional control mechanisms; however, a more likely explanation is that observed changes were the result of the primary photoreceptor damage and apoptotic loss in CAR eyes, further supporting the OCT and histology data presented here. These findings are consistent with the previous report by Grozdanic et al, who showed similar molecular and functional changes in SARDS retinas.^5^ Interestingly, while strong upregulation of different complement genes has been described in SARDS retinas, in this case the situation was not similar in CAR eyes.^5^

Observed histological and molecular changes in CAR retinas provide a unique opportunity to study numerous possible immune‐mediated responses against cancer without the background molecular noise that comes with the rapid proliferation of neoplastic cells and secondary inflammatory responses associated with neoplasia‐induced tissue necrosis and destruction. Observed immunological mechanisms in CAR retinas recapitulate some previously reported mechanisms activated during the response against cancer tissue: macrophage activation, T‐cell and B‐cell activation, and autoAb production.^48^ While CAR retinas may provide a unique opportunity to gain a better understanding of the interplay between cancer and immune system biology in terms of a tumor's destructiveness (or in the case of CAR, accidental retinal destruction), reverse thinking could be similarly applied to utilize tumor evasion strategies against the immune system to learn how to more effectively treat CAR and many other forms of AIRs.^48^

Different treatment modalities have been reported for CAR patients, which unfortunately in some cases had limited success in terms of restoring vision.^45^ All reported CAR treatment protocols are based on immunosuppression or modulation of the immune response against the retina with or without cancer reduction strategies.^45^ While the use of systemic IVIg administration has been reported for the treatment of CAR and npAIR,^49^ this is the very first study to report on therapeutic efficacy of intravitreal IVIg administration. We found it to potentially be a very safe, powerful, and cost‐effective method for treatment of many inflammatory and autoimmune ocular diseases.

Intravenous immunoglobulins have become the last resource therapy for many patients nonresponsive to traditional immunosuppressive drugs. It has been previously reported that IVIg has an inhibitory effect on autoAbs via regulation of Fab fragments, and on complement T cells, B cell, and macrophages, making it potentially an ideal candidate for the therapeutic application in npAIR and CAR patients.^50^, ^51^ In this study, we demonstrated very good clinical effect in some patients where the traditional immunosuppressive approach failed to produce a positive clinical effect. Furthermore, microarray data analysis of IVIg‐treated CAR eyes showed suppression of different immune‐mediated mechanisms, which may provide an opportunity for the wider application of this therapeutic approach for treatment of immune‐mediated and autoimmune ocular diseases. Interestingly, IVIg‐treated CAR eyes showed downregulation of activated complement components with a concurrent upregulation of complement factor H, a potent regulator of the alternative complement pathway, and complement‐mediated retinal damage.^52^ While this finding may definitely be significant for the treatment of CAR and npAIR patients, there is an interesting possibility of utilizing intraocular IVIg therapy for the treatment of age‐related macular degeneration (AMD) patients poorly responsive or nonresponsive to other forms of therapy, by nonspecific targeting of different immunological mechanisms potentially responsible for photoreceptor damage in AMD eyes.^53^

In conclusion, observed morphological and molecular retinal changes in CAR canine eyes are highly suggestive of the immune‐mediated nature of retinal damage, with many OCT structural similarities previously observed and described in human CAR patients. Better understanding of molecular events responsible for observed damage in CAR retinas may provide a unique opportunity for improving the understanding of immune‐mediated retinal damage in patients with different forms of AIRs and also may provide rare insight into the different mechanisms utilized to combat cancer development. Intravitreal IVIg application may provide a completely novel and singular opportunity for treatment of many ocular inflammatory and autoimmune diseases.

## References

[1] Van der Woerdt ANM , Davidson MG . Sudden acquired retinal degeneration in the dog: clinical and laboratory findings in 36 cases. Prog Vet Comp Ophthalmol. 1991;1:11‐18.

[2] Grozdanic SD , Harper MM , Kecova H . Antibody‐mediated retinopathies in canine patients: mechanism, diagnosis, and treatment modalities. Vet clin N Am Small Animal Pract. 2008;38:361‐387, vii. 10.1016/j.cvsm.2007.12.003 18299012

[3] Komaromy AM , Abrams KL , Heckenlively JR , et al. Sudden acquired retinal degeneration syndrome (SARDS) ‐ a review and proposed strategies toward a better understanding of pathogenesis, early diagnosis, and therapy. Vet Ophthalmol. 2016;19:319‐331. 26096588 10.1111/vop.12291

[4] Bellhorn RW , Murphy CJ , Thirkill CE . Anti‐retinal immunoglobulins in canine ocular diseases. Semin Vet Med Surg (Small Anim). 1988;3:28‐32. 3363244

[5] Grozdanic SD , Lazic T , Kecova H , et al. Optical coherence tomography and molecular analysis of sudden acquired retinal degeneration syndrome (SARDS) eyes suggests the immune‐mediated nature of retinal damage. Vet Ophthalmol. 2019;22:305‐327. 30109754 10.1111/vop.12597 PMC6563498

[6] Stromberg SJ , Thomasy SM , Marangakis AD , et al. Evaluation of the major histocompatibility complex (MHC) class II as a candidate for sudden acquired retinal degeneration syndrome (SARDS) in Dachshunds. Vet Ophthalmol. 2019;22:751‐759. 30791205 10.1111/vop.12646 PMC6703976

[7] Braus BK , Hauck SM , Amann B , et al. Neuron‐specific enolase antibodies in patients with sudden acquired retinal degeneration syndrome. Vet Immunol Immunopathol. 2008;124:177‐183. 18405980 10.1016/j.vetimm.2008.02.020

[8] Mowat FM , Avelino J , Bowyer A , et al. Detection of circulating anti‐retinal antibodies in dogs with sudden acquired retinal degeneration syndrome using indirect immunofluorescence: A case‐control study. Exp Eye Res. 2020;193:107989. 32126218 10.1016/j.exer.2020.107989

[9] Grewal DS , Fishman GA , Jampol LM . Autoimmune retinopathy and antiretinal antibodies: a review. Retina. 2014;34:827‐845. 24646664 10.1097/IAE.0000000000000119

[10] Thirkill CE , FitzGerald P , Sergott RC , et al. Cancer‐associated retinopathy (CAR syndrome) with antibodies reacting with retinal, optic‐nerve, and cancer cells. N Engl J Med. 1989;321:1589‐1594. 2555714 10.1056/NEJM198912073212307

[11] Makiyama Y , Kikuchi T , Otani A , et al. Clinical and immunological characterization of paraneoplastic retinopathy. Invest Ophthalmol Vis Sci. 2013;54:5424‐5431. 23860756 10.1167/iovs.13-11868

[12] Thirkill CE . Experimental, cancer‐induced retinopathy. Ocul Immunol Inflamm. 1997;5:55‐65. 9145694 10.3109/09273949709085051

[13] Fox AR , Gordon LK , Heckenlively JR , et al. Consensus on the diagnosis and management of nonparaneoplastic autoimmune retinopathy using a modified Delphi approach. Am J Ophthalmol. 2016;168:183‐190. 27210277 10.1016/j.ajo.2016.05.013 PMC4969197

[14] Elsheikh S , Gurney SP , Burdon MA . Melanoma‐associated retinopathy. Clin Exp Dermatol. 2020;45:147‐152. 31742740 10.1111/ced.14095

[15] Grewal DS , Fishman GA , Jampol LM . Autoimmune retinopathy and anti‐retinal antibodies. Retina. 2014;34:827‐845. 24646664 10.1097/IAE.0000000000000119

[16] Adamus G , Ren G , Weleber RG . Autoantibodies against retinal proteins in paraneoplastic and autoimmune retinopathy. BMC Ophthalmol. 2004;4:5. 15180904 10.1186/1471-2415-4-5 PMC446200

[17] Patel N , Ohbayashi M , Nugent AK , et al. Circulating anti‐retinal antibodies as immune markers in age‐related macular degeneration. Immunology. 2005;115:422‐430. 15946260 10.1111/j.1365-2567.2005.02173.x PMC1782158

[18] Cherepanoff S , Mitchell P , Wang JJ , et al. Retinal autoantibody profile in early age‐related macular degeneration: preliminary findings from the Blue Mountains Eye Study. Clin Experiment Ophthalmol. 2006;34:590‐595. 16925708 10.1111/j.1442-9071.2006.01281.x

[19] Umeda S , Suzuki MT , Okamoto H , et al. Molecular composition of drusen and possible involvement of anti‐retinal autoimmunity in two different forms of macular degeneration in cynomolgus monkey (Macaca fascicularis). Faseb J. 2005;19:1683‐1685. 16099945 10.1096/fj.04-3525fje

[20] Heckenlively JR , Aptsiauri N , Nusinowitz S , et al. Investigations of antiretinal antibodies in pigmentary retinopathy and other retinal degenerations. Trans Am Ophthalmol Soc. 1996;94:179‐200; discussion 200‐176. 8981696 PMC1312095

[21] Heckenlively JR , Jordan BL , Aptsiauri N . Association of antiretinal antibodies and cystoid macular edema in patients with retinitis pigmentosa. Am J Ophthalmol. 1999;127:565‐573. 10334350 10.1016/s0002-9394(98)00446-2

[22] Heckenlively JR , Solish AM , Chant SM , et al. Autoimmunity in hereditary retinal degenerations. II. Clinical studies: antiretinal antibodies and fluorescein angiogram findings. Br J Ophthalmol. 1985;69:758‐764. 4052361 10.1136/bjo.69.10.758 PMC1040734

[23] Romano C , Barrett DA , Li Z , et al. Anti‐rhodopsin antibodies in sera from patients with normal‐pressure glaucoma. Invest Ophthalmol Vis Sci. 1995;36:1968‐1975. 7657539

[24] Tezel G , Edward DP , Wax MB . Serum autoantibodies to optic nerve head glycosaminoglycans in patients with glaucoma. Arch Ophthalmol. 1999;117:917‐924. 10408457 10.1001/archopht.117.7.917

[25] Adamus G . Are anti‐retinal autoantibodies a cause or a consequence of retinal degeneration in autoimmune retinopathies? Front Immunol. 2018;9:765. 29713325 10.3389/fimmu.2018.00765 PMC5911469

[26] Sen HN , Grange L , Akanda M , et al. Autoimmune retinopathy: current concepts and practices (An American Ophthalmological Society Thesis). Trans Am Ophthalmol Soc. 2017;115:T8. 29576753 PMC5844289

[27] Khan N , Huang JJ , Foster CS . Cancer associated retinopathy (CAR): An autoimmune‐mediated paraneoplastic syndrome. Semin Ophthalmol. 2006;21:135‐141. 16912011 10.1080/08820530500350662

[28] Ohguro H , Yokoi Y , Ohguro I , et al. Clinical and immunologic aspects of cancer‐associated retinopathy. Am J Ophthalmol. 2004;137:1117‐1119. 15183799 10.1016/j.ajo.2004.01.010

[29] Anastasakis A , Dick AD , Damato EM , et al. Cancer‐associated retinopathy presenting as retinal vasculitis with a negative ERG suggestive of on‐bipolar cell pathway dysfunction. Doc Ophthalmol. 2011;123:59‐63. 21674222 10.1007/s10633-011-9277-y

[30] Bazhin AV , Dalke C , Willner N , et al. Cancer‐retina antigens as potential paraneoplastic antigens in melanoma‐associated retinopathy. Int J Cancer. 2009;124:140‐149. 18814277 10.1002/ijc.23909

[31] Mesiwala NK , Shemonski N , Sandrian MG , et al. Retinal imaging with en face and cross‐sectional optical coherence tomography delineates outer retinal changes in cancer‐associated retinopathy secondary to Merkel cell carcinoma. J Ophthalmic Inflamm Infect. 2015;5:53. 26285790 10.1186/s12348-015-0053-0 PMC4540718

[32] Grozdanic SD , Kecova H , Harper MM , et al. Functional and structural changes in a canine model of hereditary primary angle‐closure glaucoma. Invest Ophthalmol Vis Sci. 2010;51:255‐263. 19661222 10.1167/iovs.09-4081 PMC3258664

[33] Hernandez‐Merino E , Kecova H , Jacobson SJ , et al. Spectral domain optical coherence tomography (SD‐OCT) assessment of the healthy female canine retina and optic nerve. Vet Ophthalmol. 2011;14:400‐405. 22050777 10.1111/j.1463-5224.2011.00887.x

[34] Grozdanic SD , Matic M , Sakaguchi DS , et al. Evaluation of retinal status using chromatic pupil light reflex activity in healthy and diseased canine eyes. Invest Ophthalmol Vis Sci. 2007;48:5178‐5183. 17962471 10.1167/iovs.07-0249

[35] Ostojić J , Sakaguchi DS , de Lathouder Y , et al. Neuroglobin and cytoglobin: oxygen‐binding proteins in retinal neurons. Invest Ophthalmol Vis Sci. 2006;47:1016‐1023. 16505036 10.1167/iovs.05-0465

[36] Jiang B , Harper MM , Kecova H , et al. Neuroinflammation in advanced canine glaucoma. Mol Vis. 2010;16:2092‐2108. 21042562 PMC2965571

[37] Irizarry RA , Hobbs B , Collin F , et al. Exploration, normalization, and summaries of high density oligonucleotide array probe level data. Biostatistics. 2003;4:249‐264. 12925520 10.1093/biostatistics/4.2.249

[38] Metsalu T , Vilo J . ClustVis: a web tool for visualizing clustering of multivariate data using Principal Component Analysis and heatmap. Nucleic Acids Res. 2015;43:W566‐570. 25969447 10.1093/nar/gkv468 PMC4489295

[39] da Huang W , Sherman BT , Lempicki RA . Systematic and integrative analysis of large gene lists using DAVID bioinformatics resources. Nat Protoc. 2009;4:44‐57. 19131956 10.1038/nprot.2008.211

[40] Calderón‐González KG , Hernández‐Monge J , Herrera‐Aguirre ME , et al. Bioinformatics tools for proteomics data interpretation. Adv Exp Med Biol. 2016;919:281‐341. 27975225 10.1007/978-3-319-41448-5_16

[41] Sawyer RA , Selhorst JB , Zimmerman LE , et al. Blindness caused by photoreceptor degeneration as a remote effect of cancer. Am J Ophthalmol. 1976;81:606‐613. 179323 10.1016/0002-9394(76)90125-2

[42] Montgomery KW , van der Woerdt A , Cottrill NB . Acute blindness in dogs: sudden acquired retinal degeneration syndrome versus neurological disease (140 cases, 2000–2006). Vet Ophthalmol. 2008;11:314‐320. 19046291 10.1111/j.1463-5224.2008.00652.x

[43] Pepple KL , Cusick M , Jaffe GJ , et al. SD‐OCT and autofluorescence characteristics of autoimmune retinopathy. Br J Ophthalmol. 2013;97:139‐144. 23221966 10.1136/bjophthalmol-2012-302524

[44] Sepah YJ , Sadiq MA , Hassan M , et al. Assessment of retinal structural and functional characteristics in eyes with autoimmune retinopathy. Curr Mol Med. 2015;15:578‐586. 26238366 10.2174/1566524015666150731104626

[45] Ferreyra HA , Jayasundera T , Khan NW , et al. Management of autoimmune retinopathies with immunosuppression. Arch Ophthalmol. 2009;127:390‐397. 19365013 10.1001/archophthalmol.2009.24

[46] Carter RT , Oliver JW , Stepien RL , et al. Elevations in sex hormones in dogs with sudden acquired retinal degeneration syndrome (SARDS). J Am Anim Hosp Assoc. 2009;45:207‐214. 19723843 10.5326/0450207

[47] Leis ML , Lucyshyn D , Bauer BS , et al. Sudden acquired retinal degeneration syndrome in western Canada: 93 cases. Can Vet J. 2017;58:1195‐1199. 29089658 PMC5640279

[48] Chen DS , Mellman I . Oncology meets immunology: the cancer‐immunity cycle. Immunity. 2013;39:1‐10. 23890059 10.1016/j.immuni.2013.07.012

[49] Ramos‐Ruperto L , Busca‐Arenzana C , Boto‐de Los Bueis A , et al. Cancer‐associated retinopathy and treatment with intravenous immunoglobulin therapy. A seldom used approach? Ocul Immunol Inflamm. 2019;1‐4. 10.1080/09273948.2019.1681471 31710513

[50] Galeotti C , Kaveri SV , Bayry J . IVIG‐mediated effector functions in autoimmune and inflammatory diseases. Int Immunol. 2017;29:491‐498. 28666326 10.1093/intimm/dxx039

[51] Lünemann JD , Nimmerjahn F , Dalakas MC . Intravenous immunoglobulin in neurology–mode of action and clinical efficacy. Nat Rev Neurol. 2015;11:80‐89. 25561275 10.1038/nrneurol.2014.253

[52] Parente R , Clark SJ , Inforzato A , et al. Complement factor H in host defense and immune evasion. Cell Mol Life Sci. 2017;74:1605‐1624. 27942748 10.1007/s00018-016-2418-4 PMC5378756

[53] Toomey CB , Johnson LV , Bowes RC . Complement factor H in AMD: Bridging genetic associations and pathobiology. Prog Retin Eye Res. 2018;62:38‐57. 28928087 10.1016/j.preteyeres.2017.09.001 PMC5776047

[54] Grozdanic S , Kecova H , Lazic T .Cancer incidence in dogs with clinical presentation of Sudden Acquired Retinal Degeneration Syndrome –SARDS. Abstracts: 48th Annual Conference of the American College of Veterinary Ophthalmologists, Baltimore, MD October 11–14, 2017. Baltimore, MD: Veterinary Ophthalmology (2017) 20, 6, E1‐E19, 2017.

[55] Grozdanic S , Lazic T .Early detection of auto‐immune retinopathies (SARDS and IMR) in dogs with normal day vision. *Abstracts: 44th Annual Meeting of the American College of Veterinary Ophthalmologists, Puerto Rico, November 4–9, 2013*: Veterinary Ophthalmology (2013) 16, 6, E26‐E50, 2013.

[56] Grozdanic S , Kecova H , Chatzistefanou M , et al.Therapeutic outcomes in SARDS dogs with presence of normal day vision and completely absent retinal electrical activity. Abstracts: 47th Annual Meeting of the American College of Veterinary Ophthalmologists, Monterey, CA October 26–29, 2016: Veterinary Ophthalmology (2016) 19, 6, E21‐E43.

